# Design and commissioning of a new synchrotron beamline dedicated to X-ray footprinting mass spectrometry

**DOI:** 10.1107/S1600577526003711

**Published:** 2026-05-07

**Authors:** Sayan Gupta, Brandon Russell, Line G. Kristensen, Jared de Chant, Anthony Lu, Lieselotte Obst-Huebl, Behzad Rad, James Tyler, Simruthi Subramanian, Savannah Kidd, Sathi Paul, Yan Chen, Christopher J. Petzold, Darren N. Kahan, Shawn M. Costello, Kei Nakamura, Jamie L. Inman, Alastair A. MacDowell, Adrian Spucces, Corie Y. Ralston

**Affiliations:** a Molecular Biophysics and Integrated Bioimaging Division Lawrence Berkeley National Laboratory 1 Cyclotron Road Berkeley CA94720 USA; b Accelerator Technology and Applied Physics Division Lawrence Berkeley National Laboratory 1 Cyclotron Road Berkeley CA94720 USA; c Molecular Foundry Division Lawrence Berkeley National Laboratory 1 Cyclotron Road Berkeley CA94720 USA; d Biological Systems and Engineering Division Lawrence Berkeley National Laboratory 1 Cyclotron Road Berkeley CA94720 USA; e Biophysics Graduate Group University of California at Berkeley Berkeley CA94720 USA; f Engineering Division Lawrence Berkeley National Laboratory 1 Cyclotron Road Berkeley CA94720 USA; Australian Synchrotron, Australia

**Keywords:** X-ray footprinting mass spectrometry (XFMS), hydroxyl radical, radiation damage

## Abstract

The structural biology method of X-ray footprinting mass spectrometry (XFMS) is available at two national synchrotron beamlines in the USA: one at the Advanced Light Source (ALS) on the West Coast and the other at the National Synchrotron Light Source II on the East Coast. XFMS is a solution-state technique that utilizes oxidative modifications of proteins at micromolar concentrations in aqueous buffer to extract structural information. X-rays are employed to generate hydroxyl radicals *in situ*, which covalently modify specific protein side chains. These modifications are subsequently quantified using liquid chromatography and mass spectrometry. Ratiometric changes in modification levels between two protein states (*e.g.* with and without ligand) generate a relative solvent accessibility map of the protein pairs, which serves to reveal structural features. Up until recently, the XFMS capability was available as part of a shared program at the ALS without a dedicated beamline. In this article, we describe the commissioning of ALS beamline 3.3.1, dedicated to XFMS, including the installation of a new focusing mirror, the design and construction of a new endstation with automated sample handling and exposure capabilities, and the use of accurate empirical dose calculations using Gafchromic film. Finally, we showcase the new beamline capabilities using two protein systems.

## Abstract 2

A dedicated beamline at the Advanced Light Source synchrotron has been opened and commissioned for X-ray footprinting mass spectrometry.

## 1. Introduction

Footprinting as a tool for the investigation of biomolecular structure has a long history, with the first protein structural analysis using chemical modification methods published as early as the 1960s (Hachimori *et al.*, 1964 ▸), and the first nucleic acid–protein interactions probed using enzymatic or chemical means in the late 1970s and early 1980s (Galas & Schmitz, 1978 ▸; Van Dyke & Dervan, 1983 ▸). Studies using the term ‘protein footprinting’ were reported in the late 1980s (Sheshberadaran & Payne, 1988 ▸, 1989 ▸), with the first report using hydroxyl radicals for mapping protein–DNA interactions appearing around the same time (Tullius, 1988 ▸; Tullius *et al.*, 1987 ▸). In the latter studies, hydroxyl radicals were shown to have an advantage over enzymatic footprinting, as the hydroxyl radical (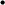
OH) is small and short-lived, leading to DNA/RNA cleavage at every base position with nearly no sequence specificity. The first reported use of synchrotron X-rays to generate 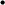
OH in the DNA and protein footprinting experiments was in 1998 (Sclavi *et al.*, 1998 ▸) and 1999 (Maleknia *et al.*, 1999 ▸), respectively, at the National Synchrotron Light Source (NSLS) at Brookhaven National Laboratory, USA. Other methods for generating hydroxyl radicals in solution have been investigated, including chemical generation such as with the the Fenton reaction (Shcherbakova *et al.*, 2006 ▸), UV laser photolysis of H_2_O_2_ (Weinberger *et al.*, 2021 ▸), electron irradiation of water (Watson *et al.*, 2009 ▸), and plasma generation (Minkoff *et al.*, 2017 ▸). A commercial system is even now available using a flash-lamp to generate hydroxyl radicals from H_2_O_2_ (Sharp *et al.*, 2021 ▸). The synchrotron-based method of X-ray footprinting mass spectrometry (XFMS) has grown in use and accessibility through the years, with a dedicated footprinting beamline established first at the NSLS in 2000 (Gupta *et al.*, 2007 ▸) and then later moved to the NSLS-II at Brookhaven National Laboratory in 2017 (Asuru *et al.*, 2019 ▸). The first synchrotron XFMS capability on the West Coast was located at the Advanced Light Source (ALS) beamlines 3.2.1 and 5.3.1, in shared use with other ALS user programs, starting in 2013 (Gupta *et al.*, 2014 ▸). The program has now been relocated to a permanent home at ALS beamline 3.3.1. Here, we describe the design, construction, and commissioning of the high-flux-density focused X-ray facility at the ALS beamline 3.3.1, dedicated to the XFMS method.

In the general implementation of the synchrotron footprinting method, a biomolecule in dilute aqueous solution is exposed to a high flux density X-ray source, and the radiolysis of water produces hydroxyl radicals *in situ*. The hydroxyl radicals modify proteins and/or cleave nucleic acids in solvent-accessible regions. The application of synchrotron X-rays for nucleic acid footprinting has been described in detail elsewhere (Sclavi *et al.*, 1998 ▸), and typically does not require high flux density except for highly complex systems. Here, we focus on protein footprinting. The solvent water absorbs most of the radiation dose for a dilute protein solution, where protein concentrations are typically maintained between 5 and 10 µ*M*, and exposure times are typically microseconds to milliseconds. Instead of direct interaction with X-rays, radiation damage to the protein is assumed to be mediated by hydroxyl radicals, which are highly reactive products of water radiolysis under such conditions (Xu & Chance, 2007 ▸). The buffer, additives, and cofactor components, which are commonly required for a protein’s native and active forms, are maintained at low concentrations, such as hundreds of micromolar to tens of millimolar concentrations, to minimize scavenging of hydroxyl radicals. The presence of highly reactive 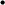
OH radical scavengers in the buffer (such as glycerol, DTT, and DMSO) can completely preclude protein side chain modification by the hydroxyl radical; hence, they are thoroughly removed by buffer exchange prior to irradiation. To empirically assess the expected extent of hydroxyl radical modification, Alexa488 fluorescence-based dosimetry is performed on the protein samples prior to the experiment (Gupta *et al.*, 2007 ▸). This step helps establish experimental feasibility, determine the optimal exposure time, and determine the appropriate X-ray exposure range.

X-ray exposure in XFMS experiments is carefully regulated to minimize radiation-induced perturbations and prevent potential conformational changes in the protein. The exposure time is controlled via a shutter for well plate-based experiments, or via sample velocity for flow-based sample exposure. In the flow-based approach, such as jet or capillary systems, samples are directly ejected into fraction collector tubes containing me­thio­nine amide, resulting in a rapid scavenging of secondary radical reactions (Gupta *et al.*, 2020 ▸). A high flux density X-ray source with X-ray focusing optics helps to prevent possible secondary oxidation reactions from reactive oxygen species other than the hydroxyl radical by reducing the exposure time to microseconds (Gupta *et al.*, 2014 ▸). Additionally, a high flux density X-ray source offers the advantage of accessing shorter timescales of structural events for time-resolved studies, as shorter overall mixing delays can be employed. After X-ray exposure, protein modifications are identified and quantified using standard bottom-up liquid-chromatography–tandem mass spectrometry (LC-MS/MS) analysis, yielding information on water positions within or at the surface of the protein, which is then used to infer structural information.

XFMS complements macromolecular crystallography (MX) and small-angle X-ray scattering (SAXS): MX provides atomic-resolution structures of proteins in the crystal state; SAXS reveals domain flexibility, rigidity, and overall molecular shape in solution; and XFMS pinpoints residue-specific solvent accessibility changes during folding and conformational transitions, identifies residue contacts in ligand binding, dynamics or complex formation, and can locate positions of bound waters which are often relevant for protein function (Gupta *et al.*, 2016 ▸; Kiselar & Chance, 2018 ▸; Gupta *et al.*, 2019 ▸; Chance *et al.*, 2020 ▸; Kristensen *et al.*, 2024 ▸; Sutter *et al.*, 2024 ▸; Martinez *et al.*, 2024 ▸). Together, MX, SAXS, and XFMS can offer a powerful and complementary toolkit at synchrotron facilities, with each method contributing unique structural data on biological systems. As of 2025, the XFMS program at the ALS is now part of the NIH P30-funded ALS-ENABLE (https://als-enable.lbl.gov) program, which partially supports the XFMS program (Ralston *et al.*, 2025 ▸).

Here we describe the design and commissioning of the dedicated ALS 3.3.1 XFMS beamline and endstation. We describe the new capabilities in the XFMS endstation, and we include empirically measured radiation doses in Gy (energy lost per unit mass, J kg^−1^ = Gy, grays) obtainable at different vertical positions in the beam focus. We showcase the new beamline capabilities using the example of XFMS analysis of the barstar–barnase complex and hybrid spectroscopy-XFMS data collection on amyloid beta aggregation in the presence and absence of Thioflavin T.

## 2. ALS beamline 3.3.1

The X-ray source at Sector 3 at the ALS is a 1.27 T bending magnet with a critical energy of 3.05 keV. The magnet feeds three beamlines: 3.2.1, 3.3.1, and 3.3.2. Beamline 3.3.2 was brought online in 2015 to serve as a general-purpose testing endstation, with an optional Si(111) monochromator (Camarda *et al.*, 2016 ▸). As part of the recommissioning of 3.3.2 in 2015, a new top-off aperture was installed in the front-end of Sector 3. The top-off aperture is located 3.7 m from the source, designed to withstand 388 W (with a peak power density of 3.28 W cm^−2^), and is currently in use for all three Sector 3 beamlines. Beamlines 3.2.1 and 3.3.1 were originally operated by the LIGA program (Griffiths *et al.*, 2004 ▸) at Sandia National Laboratory. While beamline 3.3.1 was decommissioned in 2007, beamline 3.2.1 continues to be operated by Axsun Technologies (acquired by Excelitas Technologies in 2019) for lithographic patterning of silicon substrates for microfabrication. Between 2015 and 2020, beamline 3.2.1 was also used for XFMS development and research studies. Beamlines 3.2.1 and 3.3.1 were not built with focusing optics or monochromators, and thus the raw beam size in the endstation is an unfocused broadband ∼100 mm (H) × 10 mm (V) spot. Beamline 3.2.1 remains in this configuration, while beamline 3.3.1 has been upgraded with a focusing optic as described below.

### 2.1. Mirror design and installation

**Figure 1:**
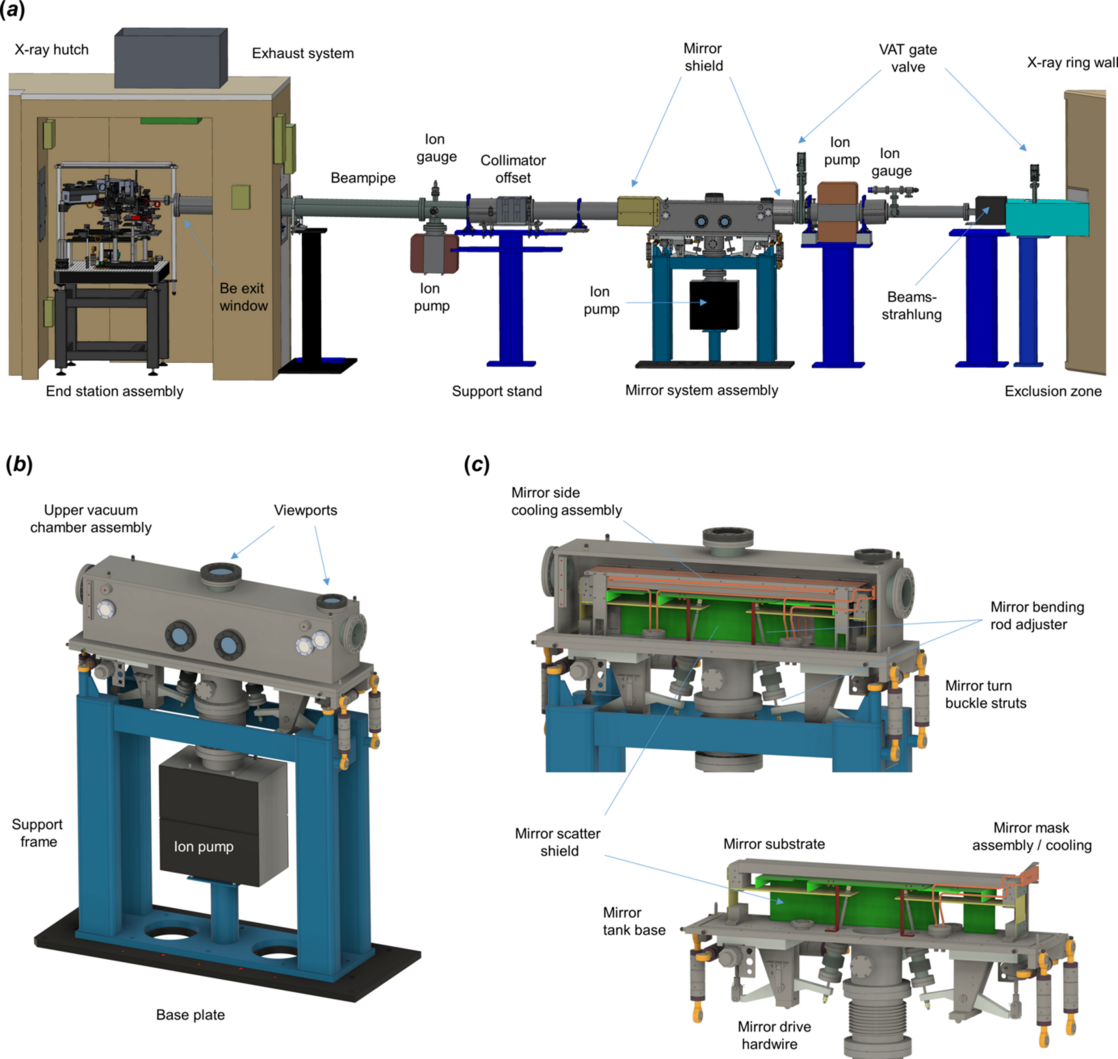
Beamline component layout. (*a*) Overview of beamline 3.3.1 components from shield wall to endstation. (*b*, *c*) Mirror assembly and its components.

For the reopening of beamline 3.3.1, an upward reflecting double focusing mirror assembly was commissioned from Advanced Design Consulting Inc. (Lansing, New York, USA). The mirror was provided by Thales SESO (Aix-en-Provence, France) [Figs. 1 ▸(*a*)–1(*c*)], with a sagittal radius of 61.6 mm, a length of 850 mm, and a sagittal width of 46 mm. The single-crystal silicon mirror was coated with rhodium for the ideal XFMS energy range of 2–10 keV and an incident grazing angle of 0.5°. Roughness was ∼4 Å RMS, with a tangential slope error of 2.22 µmrad RMS and a sagittal slope error of 3.38 µrad RMS. The new mirror tank, bend mechanisms, water cooling, and Cu mask were based on a previous design used at the ALS superbend beamlines (MacDowell *et al.*, 2004 ▸). The mirror bend mechanism produces a toroidal shaped mirror which usually requires placement at the ideal 1:1 source:sample geometry; however this was not possible due to space constraints, and the mirror was positioned 12.3 m from the source (mask at 11.8 m from the source) and the focused spot at the sample 5.45 m downstream of the mirror. The dipole source size is 94 µm (H) × 17 µm (V) FWHM, the mirror acceptance is 3.0 mrad horizontal, and the resulting focused hotspot is ray-traced to a spot size of 105 µm (H) × 197 µm (V) FWHM (Fig. S1 of the supporting information) with a long vertical tail, due to coma aberrations resulting from the asymmetric source/mirror/focus distances. We take advantage of this ‘poor’ focus by using different parts of the coma tail to vary dose rates, as described in Section 5.

The beamline layout is shown in Fig. 1 ▸. The calculated power load on the 125 µm thick Be filter upstream of the mirror is 40 W (6 mrad), with a peak power density of 0.066 W mm^−2^ at 11.5 m from the source. The 125 µm thick Be exit window is 17.3 m from the source, protrudes into the 3.3.1 hutch, and withstands atmospheric pressure on the endstation side. An interlocked nitro­gen gas flow protects this Be window from oxidation in air. The sample is typically positioned at ∼50 cm downstream of the Be window and an additional interlock prevents the shutter opening without the hutch exhaust system operating thereby preventing ozone accumulation within the hutch due to the beam ionization of air.

**Figure 2:**
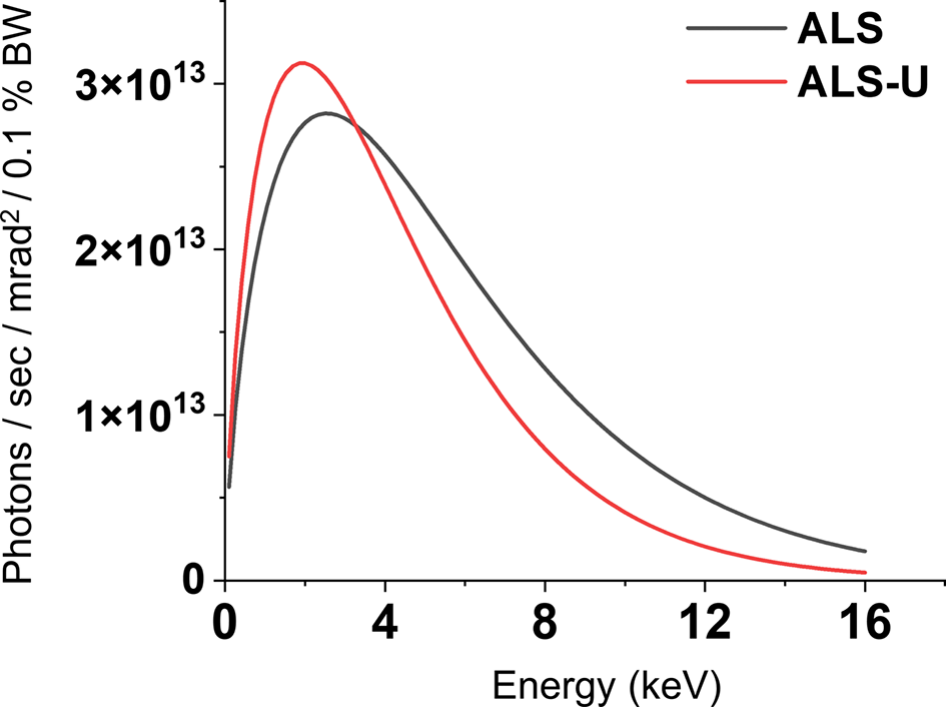
The regular bend magnets in the ALS will be replaced as part of the scheduled upgrade to ALS-U. The current bend magnet spectrum for Sector 3 is shown in black (1.27 T, 1.9 GeV, 400 mA), and the new spectrum is shown in red (0.87 T, 2 GeV, 400 mA), calculated based on the Center for X-ray Optics radiation calculator (https://henke.lbl.gov). Calculated total absorbed energy for an XFMS sample, assuming 100 µm sample depth, an air path of 12 cm, and two beamline Be windows (250 micron total thickness) will increase 1.2-fold.

The ALS is planning an upgrade (termed ALS-U) to a diffraction-limited source, with 2 GeV operation and 500 mA ring current. For the upgrade, all ring bend magnets will be replaced by lower critical energy bends (2300 eV, 0.87 T), and the repositioning of the magnets within the ring tunnel will result in the focused spot on 3.3.1 moving approximately 5 cm inboard at the sample location. In anticipation of ALS-U, the 3.3.1 mirror stand was designed with slotted holes and appropriate shielding, allowing it to be shifted laterally to accommodate the new beam position in ALS-U. The endstation in the hutch is built on an optical table that can be shifted as one unit within the hutch to accommodate the new beam exit position from the beampipe. In addition, because XFMS is a method that depends on the absorption of X-rays by water, the lower critical energy of the Sector 3 ALS-U bend magnet will lead to a slight increase in sample energy absorption (Fig. 2 ▸), which will benefit the XFMS experiment.

## 3. Endstation components and modes of data collection

Because the mirror tilt can be adjusted to position the X-ray beam focus anywhere within 5–50 cm downstream of the Be exit window within the 3.3.1 hutch, the primary components of the endstation are built on an optical table and positioned on a moveable stage, so that the sample position can be moved upstream or downstream in the hutch depending on the need for photon flux density and beam shape [Fig. 1 ▸(*a*), and Fig. S2]. However, in practice, the mirror tilt is generally left stationary, and the variation in flux density due to the focus coma tail along the vertical axis is used for various experiments, depending on the need for flux density.

The vertical sample delivery environment is designed to maximize the use of beam size and flux density along the vertical length of the focused beam. A 50–75 µm diameter liquid-jet-based sample delivery is generally used to expose samples within the high flux density hotspot with 10–50 µs exposure times, while a 200 µm inner-diameter (ID) capillary flow-based sample delivery range is generally used to expose samples in the broader vertical tail region of the beam with 150–1500 µs exposure times (Fig. S3).

A motorized X-ray slit is used to define the beam along its vertical length and reduce scattered X-ray beams for both liquid jet and capillary-based sample exposure. The sample exposure range can be further customized using a 100–200 µm diameter liquid jet, changing the vertical length of the beam using the slit, and/or using aluminium attenuation.

**Figure 3:**
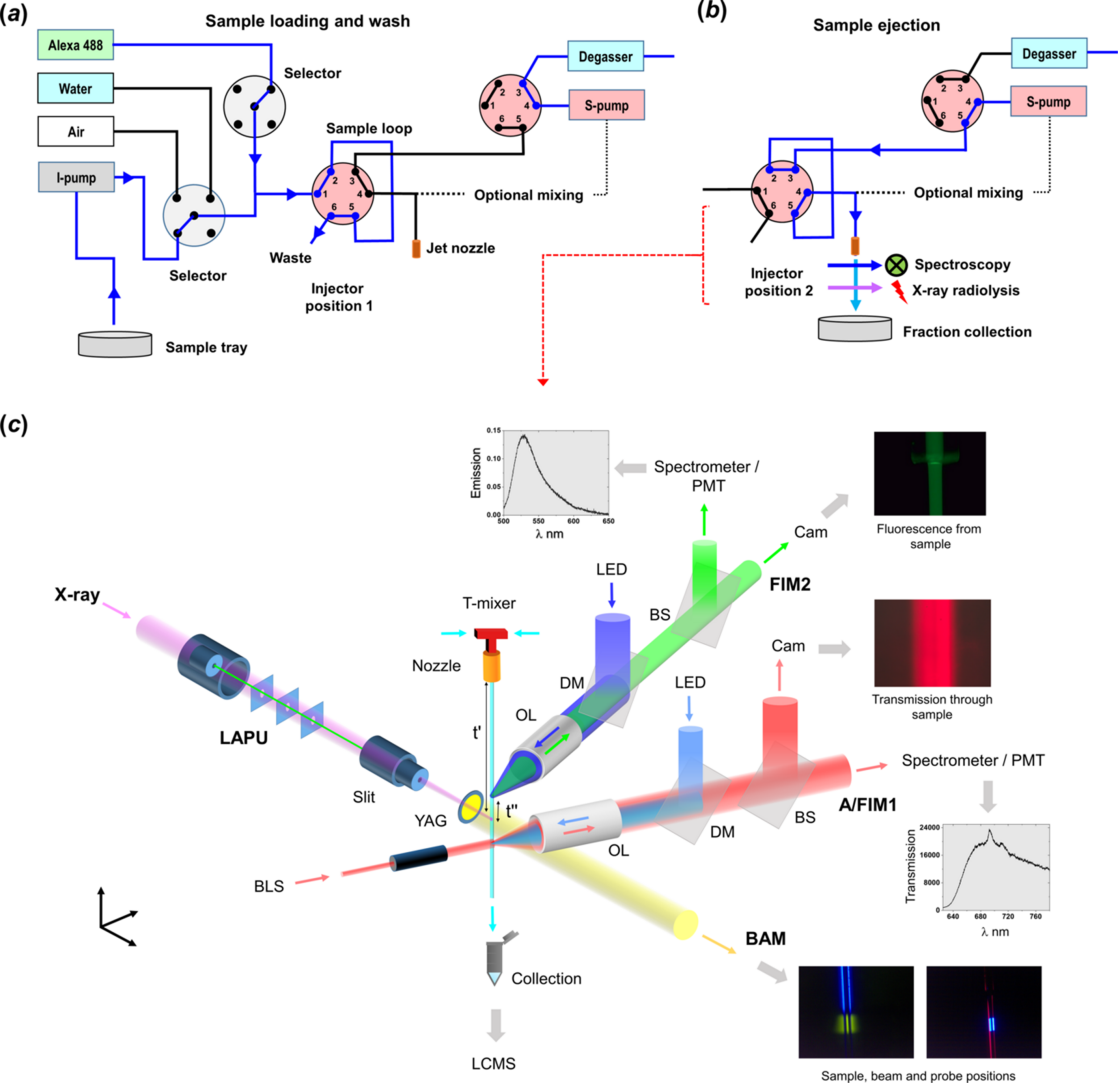
Endstation experimental components. (*a*, *b*) Schematics of the automated sample delivery system shown in two operation modes: sample loading and ejection. (*c*) Layout of the components for beam-alignment and hybrid spectroscopy-XFMS data collection. Inserts are examples of spectroscopy data from the spectrometer and sample images from the imaging modules.

The endstation instrument contains three main modules: the sample delivery module, a beam alignment module (BAM), and absorption/fluorescence imaging modules (A/FIM). Each module consists of numerous interchangeable components and is mounted on a motorized stage, allowing for precise alignment with supplemental manual control (Fig. 3 ▸).

### 3.1. Beam alignment module

The BAM is an in-house, innovative, high-precision alignment module that combines captured X-ray fluorescence from a diamond or neodymium-doped yttrium aluminium garnet (Nd:YAG) screen, jet/capillary edge illumination utilizing the total internal reflection properties of the liquid column, and high-resolution image recognition with a center detection algorithm, as previously described for live alignment (Gupta *et al.*, 2025 ▸). This module also features a laser-assisted pre-alignment unit (LAPU) with a three axial aligned pinhole, which helps bring the micrometre-sized focused X-ray beam within <700 µm of the jet stream more efficiently than conventional two-dimensional beam profile scans.

### 3.2. Sample delivery module

The original design of the sample delivery module consisted of a high-pressure syringe pump that generates a high flow velocity to either eject the sample through a jet nozzle or flow the sample through a capillary (Rosi *et al.*, 2022 ▸). The samples are manually loaded into either a 1 ml or 2.5 ml Hamilton 1000 series gas-tight syringe; the minimum sample exposure volume is 25 µL, which is collected in the fraction collector. The new features include a fully automated microfluidic sample handling and delivery system consisting of several VICI four-port selectors and high-pressure six-port injector valves for sample selection, the optional mixing of Alexa with sample for fluorescence dosimetry, sample loading in the sample loop, and a high-pressure hydraulically driven variable-size sample jet for sample ejection and irradiation. The sample delivery system features a reservoir with accompanying pumps, enabling automatic washing of the capillary and tubing after sample exposure [Figs. 3 ▸(*a*) and 3(*b*)].

### 3.3. Imaging modules

The Fluorescence Imaging Module (FIM) can be used for hybrid data collection mode, as described previously (Gupta *et al.*, 2025 ▸). In this mode, collimated excitation light from the LED is directed through a dichroic mirror (DM) and a long-working-distance objective lens (OL) to excite the sample both above and below the X-ray impingement point, enabling simultaneous pre- and post-exposure spectroscopy coupled to XFMS. Fluorescence emission is collected through the same lens, filtered, and split by a beam splitter (BS), where part of the emission goes to a photomultiplier tube (PMT) coupled with a solenoid shutter or IsoPlane 160, coupled with a ProEM 1600×200 EMCCD detector for spectral analysis. At the same time, the remaining light is sent to a camera (Cam) for real-time monitoring. A new feature includes absorption spectroscopy using the Absorption Imaging Module (AIM), where a high-magnification (50×) objective lens is used to collect data from an area inside the 100–200 µm ID sample to cut off any background incident light from a broadband light source (BLS). Similar to the design of the FIM, the collimated LED light in the AIM, which is used for alignment, is directed through a dichroic mirror and then through the objective lens. The BAM and A/FIM facilitate quick alignment and data collection from the sample at any position. Images from the A/FIM and BAM are analyzed using *ImageJ* (Schneider *et al.*, 2012 ▸) for optomechanical measurements, including beam and sample size, delay time, beam profiles, and FWHM estimation, among other parameters. Currently, only one FIM and one AIM module is integrated into the endstation setup. However, the design enables the accommodation of more imaging modules for multimodal spectral analysis above and below the X-ray exposure point, utilizing various light sources, optics, and detectors for simultaneous spectroscopy [Fig. 3 ▸(*c*)].

### 3.4. Time-resolved capabilities and software control

The beamline is equipped with a new stopped-flow mixing configuration designed for hybrid XFMS with inline spectroscopy with near 10 ms mixing delays, providing time-resolved studies for simultaneous global and local structural analyses across key bimolecular time regimes. In the updated design, the T-mixer is connected to the sample loops of the six-port injector valves, and mixing and ejection are carried out by a high-pressure, hydraulically driven system capable of withstanding the back-pressure of a high-speed 50 µm sample jet, providing submillisecond mixing time with single-digit microsecond X-ray exposure. A LabVIEW-based software interface is used to set flow parameters, select mixing mode and delays, execute sample exposure and fraction collection, and collect pre- and post-exposure fluorescence data for inline spectroscopy and Alexa-based dose-response analysis (Fig. S4). The timing of the sample flow and mixing, X-ray exposure, and simultaneous collection of fluorescence emission data are critical aspects of the automation. Details on flow parameter considerations have been described previously (Ralston *et al.*, 2025 ▸).

### 3.5. Sample preparation, post-exposure sample processing, and data handling

Post-exposure, samples can be stored at −80°C, sent to a user institution, or immediately prepared for LC-MS/MS analysis. A ThermoFisher Orbitrap Exploris 480 LC-MS instrument, housed in an adjacent lab, is now used for characterizing the majority of XFMS samples run on beamline 3.3.1. Typical LC-MS data collection parameters have been described previously (Gupta *et al.*, 2020 ▸). Data from the Orbitrap are transferred to a remote-accessible server hosting the Protein Metrics *Byos* software, so that users and staff can begin data processing immediately after LC-MS/MS acquisition remotely.

## 4. Estimation of dose rate by Gafchromic film dosimetry

Due to the nature of the focused beam, the dose absorbed by a sample will be a function of the spatially varying dose rate profile of the beam and the time the sample spends in each region. By utilizing a defined beam size, either through a slit or a specific sample diameter, or both (discussed further in Section 5), the dose rate applied to the sample can be controlled by isolating discrete bands of the beam for the sample to pass through.

The Alexa488 dye is commonly used as a chemical dosimeter in XFMS experiments to estimate the relative hydroxyl dose for various buffers and protein samples (Gupta *et al.*, 2007 ▸; Gupta *et al.*, 2010 ▸; Rosi *et al.*, 2022 ▸). Although the development of microfluidic-based automated Alexa488 dose-response analysis now provides a rapid and efficient way to assess relative dose levels, an accurate empirical dose measure is vital for radiation damage studies in which the actual dose absorbed in the Gy scale can be correlated with exposure conditions. This is particularly necessary for studies on radiation damage to biomolecules or cell samples where heavily attenuated X-ray beams with low flux density are used. For example, in previous dose rate studies, irradiation was carried out in the presence of 1–2 mm aluminium attenuation, resulting in low enough flux density that chemical dosimetry, such as Alexa dosimetry, would not be sensitive enough (Gupta *et al.*, 2023 ▸).

**Figure 4:**
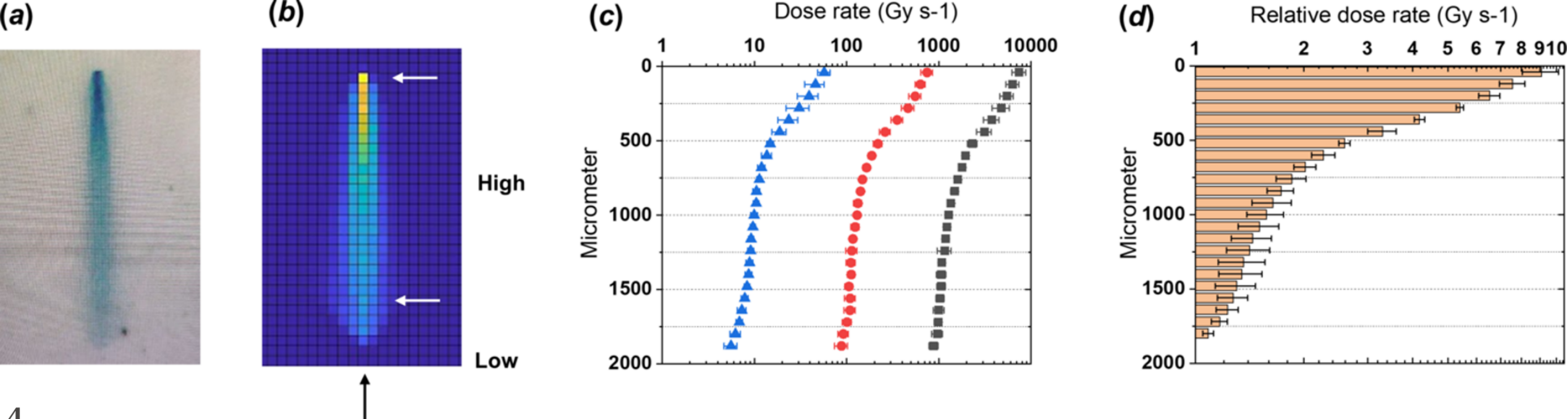
Dose estimation by Gafchromic film dosimetry. (*a*) An overexposed Gafchromic film illustrating full beam shape at the mirror focus. (*b*) Visualization of radiochromic film (RCF). The RCF was saturated for this exposure time (2 s) and attenuation (0.5 mm Al). Dose rate analyses were carried out for each region of approximately 80 µm × 80 µm between the two white arrows along the center of the vertical axis of the beam shown by the black arrow. (*c*) Dose rates at various Al attenuation levels, 0.5 mm, 1.0 mm and 2.0 mm shown in black, red and blue, respectively, along the center of the vertical axis of the beam between the two white arrows shown in (*b*). (*d*) Relative dose rate at the Al attenuation levels and positions shown in (*b*).

To characterize the 2D spatially varying dose rate, dose measurements were performed using Gafchromic HD-V2 radiochromic film (RCF), which is widely used in radiation therapy for measuring radiation doses (10–1000 Gy) (Niroomand-Rad *et al.*, 2020 ▸). The active layer of the RCF is sensitive to ionizing radiation. It undergoes a color change upon exposure, and the degree of darkening, or optical density (OD), of the film can be correlated to the absorbed dose [Fig. 4 ▸(*a*)]. The films were placed at the focal position of the X-ray beam in place of the liquid jet and irradiated with various exposure times using a Uniblitz XRS6 X-ray shutter (Vincent Associates, NY, USA) and with varying levels of attenuation (0.5 mm, 1 mm, and 2 mm of Al, 0.03–18 s exposure time; Table S1 of the supporting information) to characterize the dose rate profile of the beam. Irradiation of the films without attenuation could not be performed due to the risk of damage to the shutter and overexposure of the film with the minimum shutter speed of 10 ms. Films were scanned using an EPSON Expression 12000XL scanner in landscape format, with all image correction features turned off, at a resolution of 600 dpi (42.3 µm pixel^−1^) in transmission mode. The scanned images were saved as 16-bit grayscale TIFF files. Scanning was conducted several days after irradiation to allow for stabilization of the optical density development post-irradiation. The pixel values of each scan were converted to OD calibrated using a NIST-calibrated transparent step wedge, which can be directly correlated to the measured dose. The conversion from OD to dose delivered to the film was performed according to the high-dose-rate calibration of HD-V2 films (Bin *et al.*, 2019 ▸).

To obtain the dose rate profile of the beam, the dose measured in each pixel in an RCF scan was divided by the exposure time to calculate the dose rate measured in each pixel. Approximately 84 µm in width (two pixels) was used to estimate the dose applied to the samples in the 75 µm-wide liquid jet along the vertical center axis of the focused beam [Fig. 4 ▸(*b*)]. To convert the dose measured by the film to the dose applied to the aqueous sample, Monte Carlo (MC) simulations using *TOPAS* (Perl *et al.*, 2012 ▸) were performed with the full X-ray spectrum and beamline geometry incorporated. The simulations modeled the energy deposition in the HD-V2 active layer (chemical composition per Niroomand-Rad) and compared it with the dose deposited in 75 µm of water. A conversion factor was determined as
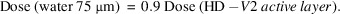
This implies that the dose directly measured by the film underestimates the dose applied to the sample in the liquid jet. The dose rate values shown in Fig. 4 ▸(*c*) and Table S1 are based on the film measurements and are scaled by this factor to account for the difference in absorption between the HD-V2 active layer and water. While the spectrum of the X-ray beam does vary with different levels of attenuation, this factor was found to be consistent across MC simulations with the three thicknesses of aluminium present in the beamline. The dose rates for each 80 µm × 80 µm region of the beam showed nearly ten-fold variations and roughly corroborated the beam ray trace [Fig. 4 ▸(*c*), Table S2]. Prior studies have reported that Gafchromic RCF of the model EBT3 exhibits an intrinsic energy dependence and under-responds at low photon energies, with up to 20% at mean photon energies < 15 keV when compared with a ^60^Co source (Hammer *et al.*, 2018 ▸). This under-response appears to be independent of the difference in absorption between film and water and is inherent to the chemical mechanism underlying the dose–OD response. While a similar investigation at energies relevant to the ALS 3.3.1 beamline with HD-V2 films is not found in the literature, due to the similar chemical composition between the models, it is likely that HD-V2 also under-responds to low-energy photons compared with higher-energy photons. Consequently, caution should be exercised when comparing the measured dose rates in this facility with those in other facilities. Further validation of the dose rate of the beamline using alternative dosimetry techniques, such as low energy calibration ion chambers and semiconductor dosimeters, is a subject for future work.

## 5. Sample exposure considerations at various locations of the beam by Alexa488 dose-response analysis

**Figure 5:**
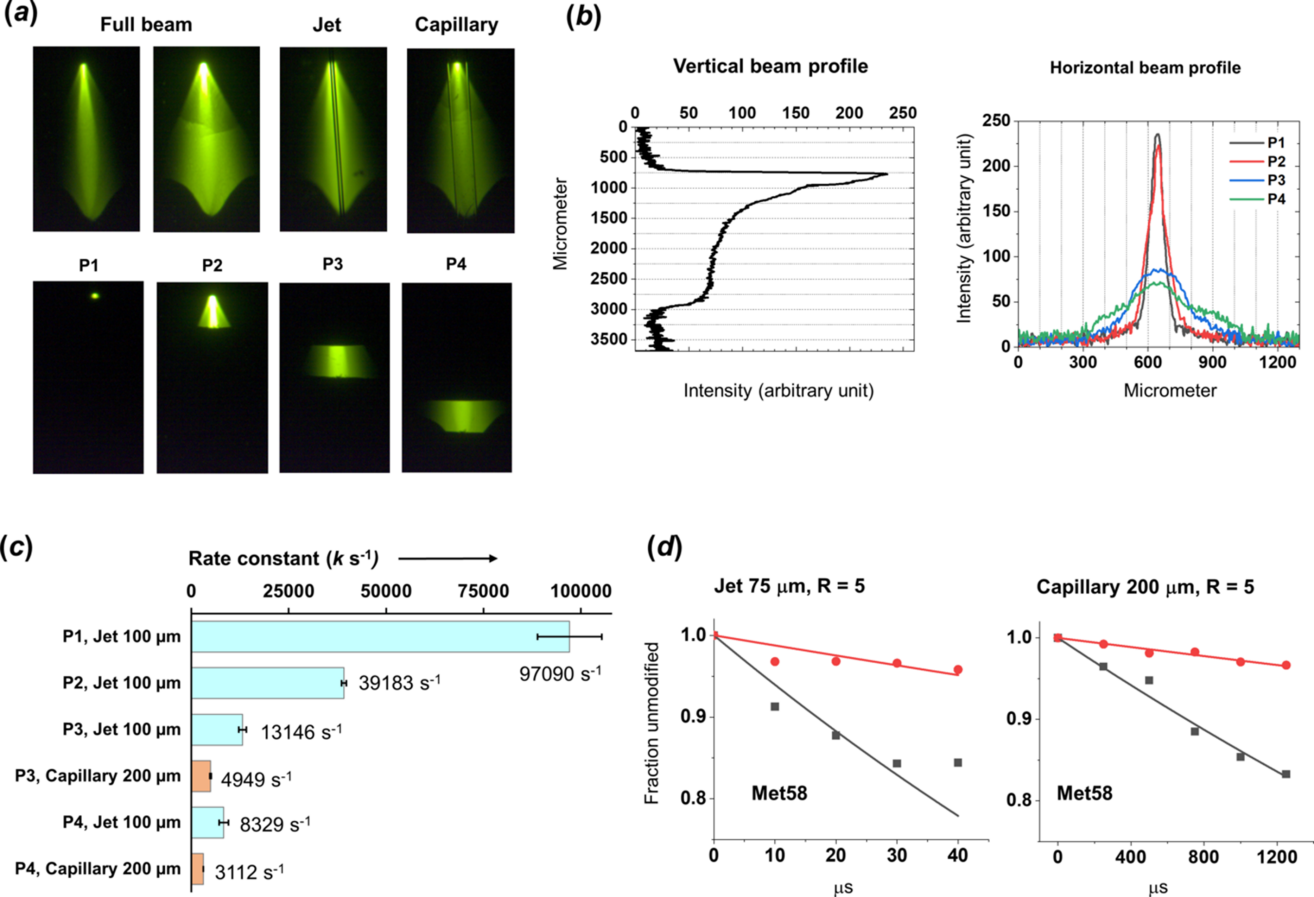
Experimental dose calculation. (*a*) The top panel shows images of the beam and the shadows of the 100 µm jet and 200 µm ID capillary on the Nd-YAG screen as captured by the BAM. The bottom panel shows the segmented beam using a 460 µm vertical X-ray slit at the four selected positions, P1 to P4. (*b*) Vertical and horizontal beam intensity profile determined using *ImageJ* for the full and truncated beam, respectively. (*c*) Hydroxyl radical reactivity rate for Alexa488 exposed at positions P1 through P4 using the 100 µm jet and the 200 µm ID capillary. (*d*) Example of dose response data of residue M44 using the 75 µm jet and 200 µm ID capillary for free SpyCatcher003 (black) and SpyCatcher003–SpyTag001 complex (red). Both jet and capillary flow showed a similar ratio of modification (*R*) at residue M44 upon complex formation, where *R* represents the ratio of the hydroxyl radical reactivity rate between the free and complex states.

The XFMS sample exposure configuration is established by jet or capillary flow to achieve optimum exposure as described previously (Gupta *et al.*, 2020 ▸). In a typical XFMS experiment, a series of samples with progressively increasing dose is collected to generate a dose-response plot. The X-ray flux density is the key factor that determines whether the user should use the jet or the capillary to collect the series of samples. Based on the beam shape observed by BAM and subsequent FWHM measurement using *ImageJ*, we isolated four sections (P1 to P4) using a motorized X-ray slit and tested their usability for XFMS sample exposure. Section P1 was 60 µm in height and included just the hotspot at the top of the beam, while P2, P3, and P4 were each 460 µm in height, isolated from various positions of the beam [Fig. 5 ▸(*a*)]. Because of the differences in beam homogeneity in terms of flux density at the different positions, we used a 100 µm jet for all four positions, but, in addition, a 200 µm ID capillary for the P3 and P4 positions. Capillary and jet positions were aligned along the central vertical axis of the beam using motorized stages based on their FWHMs [Fig. 5 ▸(*a*) and 5(*b*)].

Alexa488 fluoro­phore was prepared at its standard XFMS concentration of 2 µ*M* in 10 m*M* phosphate buffer at pH7. Inline fluorescence was collected as previously described to obtain Alexa488 dose rates for each position using capillary and jet modes, with exposure times ranging from 250 to 1000 µs and 6 to 50 µs, respectively, with 25 µm aluminium attenuation. Position P1, located at the hotspot, delivered the highest flux density, resulting in an Alexa488 rate of ∼97000 s^−1^ [Fig. 5 ▸(*c*)]. From a radiolytic labeling standpoint, position P1 is comparable with the dose delivered by the focused beam spots at beamline 17 BM of NSLS-II and beamline 5.3.1 of the Advanced Light Source. Region P1 is used for single to low double-digit microsecond exposures with a jet diameter of up to 100 µm. The longer path length of P2, which includes the hotspot, is advantageous when extended exposure times (up to 100 µs) are needed without significant loss in total dose or flux density. Such extended exposure is not possible with a short vertical path, such as that of P1, as the delivery flow rate falls outside the jetting velocity. The P2 region, which exhibits an Alexa488 rate of ∼40000 s^−1^, falls within the jetting velocity range and can still be utilized for XFMS sample exposure, considering that there is significant dose averaging due to the inhomogeneous nature of the flux density along the sample delivery path. Neither P1 nor P2 is suitable for a 200 µm ID capillary because of the narrow horizontal FWHM. The flux densities at positions P3 and P4, which showed Alexa488 rates of ∼13000 s^−1^ and 8000 s^−1^, respectively, are not suitable for high-velocity jet-based sample delivery because the exposure times are too short to produce an adequate amount of modification. Since capillary flow can utilize a low flow rate, sample exposure at these two positions provides a sufficient amount of modification in hundreds of microseconds. The Alexa488 dose rate at P3 and P4, when using a capillary, would be ∼2.5-fold lower due to X-ray absorption by the glass, as reported earlier (Gupta *et al.*, 2020 ▸).

We tested the usability of positions P2 and P3 using the SpyCatcher003–SpyTag001 split protein system, a bioengineered protein (SpyCatcher) and peptide (SpyTag) pair that can covalently bind to each other and form a stable complex through an isopeptide bond (Keeble *et al.*, 2019 ▸). We previously used a capillary setup for detailed studies on solvent accessibility changes in the residues that are either directly involved or in proximity to the binding interface (Gupta *et al.*, 2025 ▸). One of the residues, M44, located at the interface, showed ∼5-fold protection upon covalent complex formation between SpyTag003 and SpyCatcher001. Here, using a jet for exposures in the tens of microseconds at position P2 gave a similar level of protection as the capillary-based exposures in the millisecond range at P3 [Fig. 5 ▸(*d*)], indicating that both P2 and P3 can be used for sample exposure to provide the same change in solvent accessibility information. However, the jet sample delivery can provide a significant temporal advantage for time-resolved inline hybrid spectroscopy-XFMS studies, and offers an advantage when shortening exposure time is necessary to minimize sample perturbation. Although the Gafchromic film dosimetry (Section 4) roughly estimated that position P1 receives a higher amount of homogeneous flux density relative to P2, where the flux density varies 4–5-fold [Fig. 5 ▸(*c*)], the ease of using a larger area of P2 outperformed the disadvantage of using P1 for jet-based exposure to achieve even shorter microsecond exposures. Overall, the jet-based Alexa488 dosimetry results roughly correspond to the relative dose rates estimated by Gafchromic film dosimetry at the selected sample exposure regions. The Alexa488 dosimetry was performed with short exposure but high flux density to control the effect of secondary radiation damage, while Gafchromic film dosimetry was performed with a long exposure and an attenuated beam to prevent overexposure of the film. A direct correlation between Alexa488 and Gafchromic film dosimetry might be possible if the range of exposure times is similar for both methods, which could be achieved by using either a fast microsecond shutter or a fast translation stage.

## 6. Considerations for single and multipoint-based XFMS data collection

In the standard multipoint XFMS workflow, a series of exposures are collected in order to create a dose-response plot, which is then fitted with a single exponential decay to calculate the pseudo-unimolecular hydroxyl radical reactivity rate constant (Gupta *et al.*, 2016 ▸). Under a fixed flux density or dose rate of the X-ray beam, the concentration of the reactive species, 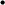
OH, is assumed to be constant throughout the duration of each exposure time, and the progressive increase in exposure time generates increasing side chain modifications without any loss or change of the steady-state 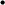
OH radical concentration. The rate constants calculated from the dose-response plot for each peptide or residue reflect both the solvent accessibility and intrinsic reactivity of that particular peptide or residue. The ratio of hydroxyl reactivity rates between two protein states is used to effectively normalize out the intrinsic reactivity, allowing solvent accessibility changes between the different states to be quantified. In addition, the multiple points within the dose-response serve as dose rate replicates (Gupta *et al.*, 2025 ▸).

Here, we have investigated conducting the XFMS experiment using repeats of one single exposure, as is done in the fast photochemical oxidation of proteins (FPOP) method (Johnson *et al.*, 2019 ▸). We used the Alexa488 fluorescence assay in the presence of a protein to estimate errors associated with single-point sample exposure at various flow velocities (or exposure times) (Fig. S5) for the capillary flow method. For a given exposure time, the error related to any fluctuation or instability of the sample or beam is estimated to be low for the following reasons: First, the exposure volume on the sample path at the exposure window is negligible (1–20 nL) compared with the total exposed sample volume (50–200 µL). Therefore, for a given dose or exposure time, each collected sample is effectively an average of many smaller samples. Second, the FWHM of the beam is set to 1.5 to >3 times wider than the sample width, contributing to the low exposure error associated with any vibration, which was within 10–20 µm for the liquid jet and nearly zero for capillary flow-based sample exposure detected by the FIM during sample exposure. However, whenever permitted, it is advisable to consider sample batch replicates and/or post-exposure sample processing and LC-MS technical replicates. Single-point analysis usually has lower sample volume requirements than multipoint analysis. However, single-point analysis requires that a sufficient dose be used to yield adequate modification for quantitative analysis. Unlike multipoint analysis, an inadequate or over-exposed sample can lead to underestimation of the observed change between two samples. In theory, both multipoint and single-point analysis should lead to the same conclusion regarding solvent accessibility changes.

**Figure 6:**
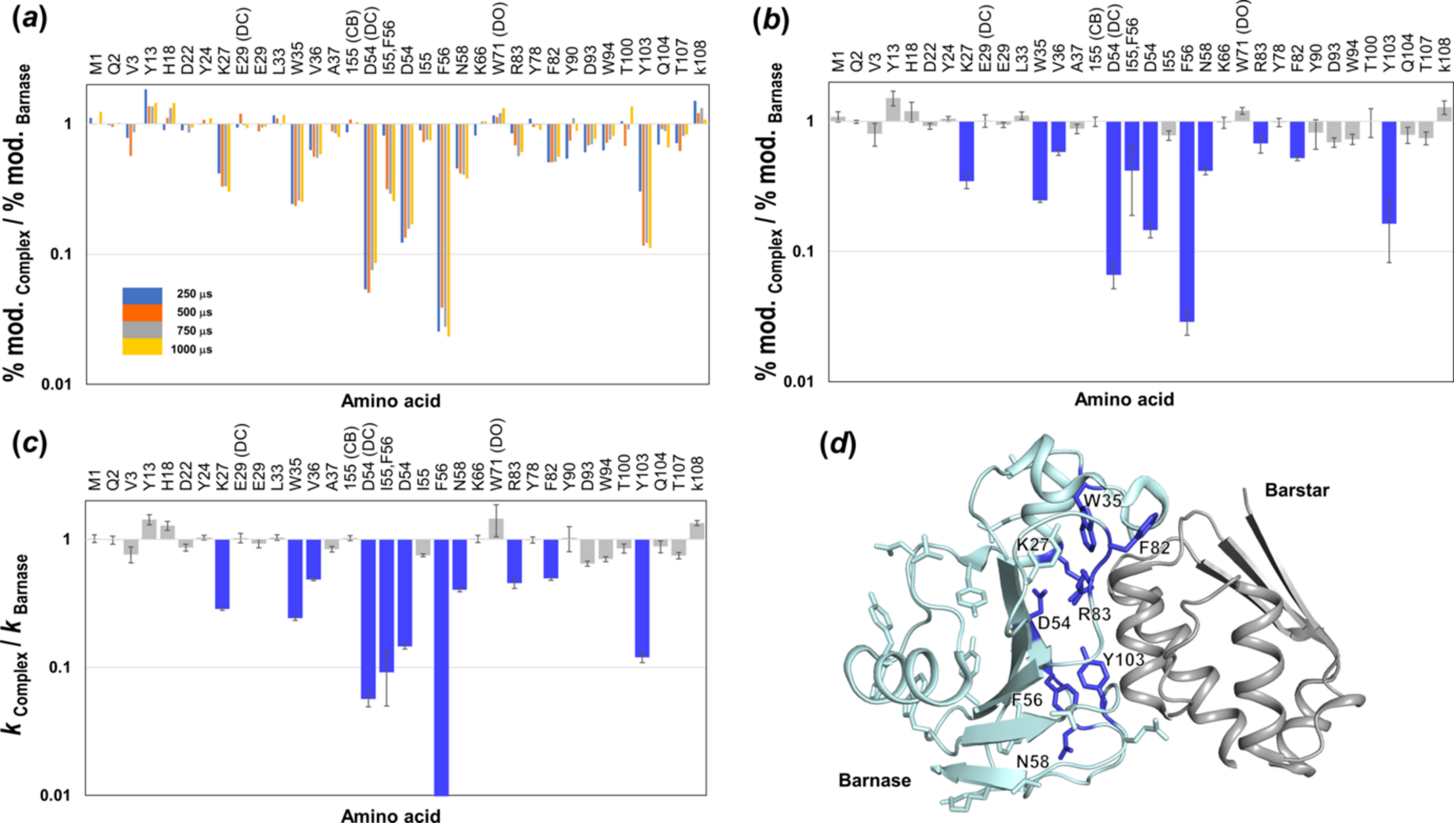
Single versus multiple exposure approach. (*a*) Bar plot showing ratio of % modification of amino acid residues at each exposure between the barstar–barnase complex and free barnase. (*b*) Bar plot generated by the % modification analysis showing either decrease (blue) or no change (gray) in solvent accessibility of amino acid residues in the barstar–barnase complex. The error bars show standard deviation for the dose rate replicates shown in Table S4. (*c*) Bar plot generated by XFMS dose-response analysis showing either a decrease (blue) or no change (gray) in solvent accessibility of amino acid residues when the barstar–barnase complex is formed. The error bars show the maximum and minimum values of the ratios shown in Table S3. (*d*) Residues of barnase highlighted with blue indicated >2-fold decrease in solvent accessibility upon complex formation. The structural model was generated by PDB 2za4 (Urakubo *et al.*, 2008 ▸).

To compare results between multiple versus single exposure approaches, we performed XFMS of the barstar–barnase complex (Buckle *et al.*, 1994 ▸). The barstar and barnase proteins were purified (see Method S1 of the supporting information), and the free barnase protein and the barstar–barnase complex were exposed to a progressive increase in dose at a fixed dose rate. For the single point exposure analysis, we compared the ratio of % modification at each dose as well as the average dose between complex and free barnase protein [Figs. 6 ▸(*a*), 6(*b*), and Table S4]. For the multipoint dose-response-based approach, we determined the hydroxyl reactivity rate and the ratio of rates between complex and free samples [Fig. 6 ▸(*c*), and Table S3]. From the ratio, we identified the specific residues involved in binding interactions [Fig. 6 ▸(*d*)]. In this example, the protein interaction sites as determined by the relative changes in the solvent accessibility by both approaches did not show any significant differences. However, we note that the single point approach might generate less solvent accessibility differences when the sample is overexposed. Overexposure usually damages samples, initiates secondary radical chain reactions, and leads to a nonlinear or flat dose-response. The multipoint approach has the advantage that overexposure becomes obvious when fitting the data, and the last (overexposed) point can be dropped, whereas with the single point measurement the user might not be aware that the sample was overexposed. In general, using a multipoint dose-response approach has the advantage of generating less error; however, the difference might not be significant if other technical replicates are used for each exposure point, and when doses are optimized carefully. Neither approach therefore necessarily reduces the number of LC-MS runs, although single point analysis will consume less sample when a flow-based setup is used. One advantage of the new beamline endstation configuration is that random exposure errors originating from beam current fluctuation have been reduced by introducing auto-logging of beam current values in the exposure metadata, and sample misalignments are minimized by introducing a live sample alignment view through the BAM and A/FIMS modules. In addition, users are recommended to routinely check for sample deposition and clogging in the tubing and exposure cell to reduce generating systematic errors during data collection.

## 7. Monitoring the Aβ aggregation pathway by simultaneous fluorescence and XFMS

In recent years we have developed a hybrid experimental mode of data collection that integrates fluorescence-based global structural analysis with X-ray hydroxyl radical footprinting, and evaluated the feasibility and utility of combining these two orthogonal methods in real time (Gupta *et al.*, 2022 ▸; Gupta *et al.*, 2025 ▸). Here we report another application of the hybrid spectroscopy-XFMS approach to study amyloid β (Aβ) peptide aggregation. Aβ of various lengths is produced via enzymatic cleavage of amyloid precursor protein (APP) and has been implicated in Alzheimer’s Disease (AD) due to its aggregation into oligomers and fibrils found in brain plaques. However, the role of Aβ aggregation in AD progression remains an active area of investigation (Jeremic *et al.*, 2021 ▸). Structural studies using techniques such as cryoEM, NMR, and biophysical assays have provided insights into fibrillar forms of the peptide (Colvin *et al.*, 2015 ▸; Kollmer *et al.*, 2019 ▸; Dresser *et al.*, 2021 ▸), and structural mass spectrometry methods like FPOP, XFMS, and hydrogen deuterium exchange mass spectrometry (HDX-MS) have yielded some information on residue or peptide-specific solvent accessibility and flexibility during Aβ aggregation (Klinger *et al.*, 2014 ▸; Li *et al.*, 2016 ▸; Zhang *et al.*, 2013 ▸).

**Figure 7:**
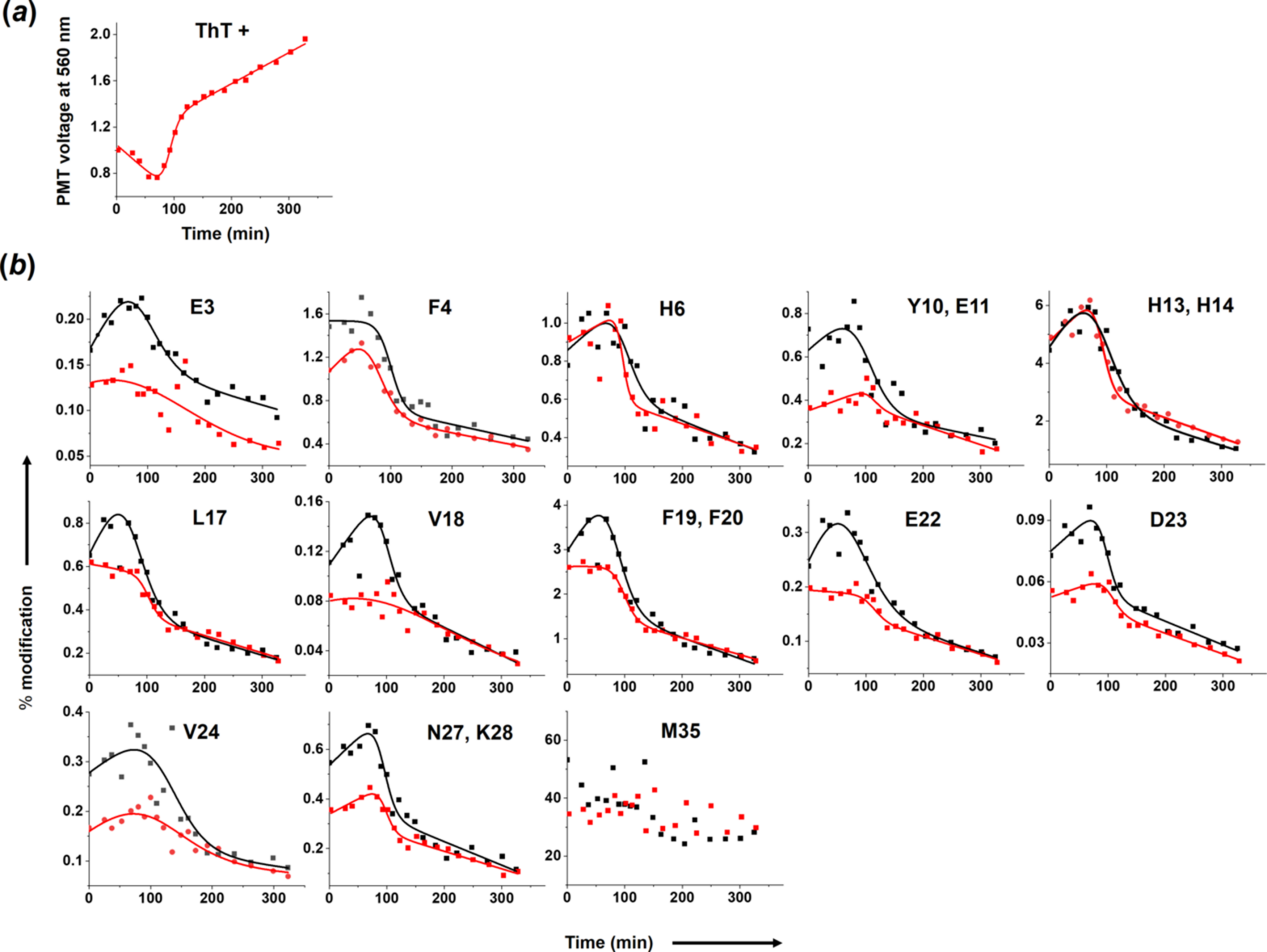
Monitoring Aβ40 aggregation by near-simultaneous fluorescence and XFMS. (*a*) In-line fluorescence collected in the presence of ThT in Aβ40 at various delays after the initiation of aggregation by solvent transfer (see Method S2). (*b*) The kinetic traces of residue level modification in the presence (red) and absence (black) of ThT were obtained by exposing the sample to X-rays in the standard XFMS experiment 100–500 µs after in-line fluorescence collection. The solid line represents fitted data using a modified Boltzmann sigmoidal equation (Table S5).

Characterizing the aggregation of this peptide is challenging in part because aggregation is notoriously dependent on physical factors like temperature, vibration, pressure, *etc*., and chemical factors such as the solvent, additives, and other protein and peptide components. Because of this, a common method to follow aggregation is the well known Thio­flavin T (ThT) fluorescence assay. ThT binds specifically to β-sheet-rich structures characteristic of amyloid fibrils, resulting in a significant increase in fluorescence intensity with the rise in fibril formation [Fig. 7 ▸(*a*)]. Although the ThT assay provides an overview of the biphasic phase transition of Aβ aggregation for any type of solution-based assay, it lacks information at the residue level on the polypeptide undergoing aggregation.

Here, we characterized the 40-mer form of Aβ by leveraging the ThT assay protocol to track fibril formation in real time at beamline 3.3.1, while simultaneously monitoring the site-specific kinetics of solvent accessibility of the Aβ40 peptide. Inline integration of microfluidic-based laser-induced fluorescence, approximately 100 to 500 µs (depending on the exposure time or sample flow rate) prior to X-ray irradiation, permits near-simultaneous collection of global structural data from ThT fluorescence and residue-specific information from X-ray irradiation and LC-MS analysis (Fig. 7 ▸, Method S2). We monitored two parallel samples of aggregating Aβ, one with and the other without ThT using the same microfluidic configuration with an approximate delay of 3 min, which is necessary for completing data collection for each type of sample. The delay was incorporated in the kinetic traces. We compared Aβ40 aggregation with and without ThT under identical conditions by monitoring the % modification, which is proportional to solvent accessibility and intrinsic reactivity, at the residue level as quantified from LC-MS/MS analysis of single point X-ray irradiation [Fig. 7 ▸(*b*)]. The overall modification level dropped after the transition midpoint, which is consistent with the formation of protein aggregates by exclusion of surrounding water. Although we observed biphasic kinetic pathways with a similar midpoint of transition (*t*_50_, Table S5) for both sets of data for most residues, there were noticeable differences in the slope as well as the values of the rate of solvent accessibility changes before the *t*_50_ point. Residues Glu3, Tyr10/Glu11, Leu17, Val18, Phe19/Phe20, Glu22, Asp23, and Asn27/Lys28 showed an indication of the formation of the intermediate state before the *t*_50_ point and with a higher degree of solvent accessibility in the ThT free state, compared with the ThT bound counterpart. In contrast, the difference is less pronounced for Phe4, His6, and His13/His14 residues, which showed similar kinetic traces for both ThT free and bound states. We emphasize here that, because of the dependence of Aβ aggregation on many physical parameters, these results are representative only, and serve to highlight the utility of using simultaneous fluorescence and XFMS characterization. This result suggests that ThT is interacting with non-fibrillar forms of Aβ40, though this would have to be confirmed by XFMS dose-response analysis throughout the progression of the aggregation, assuming that the aggregation speed is slow relative to the speed of carrying out the dose-response data collection at each time point. A detailed discussion is not the goal of the present study, which is focused on showcasing the capabilities of the beamline 3.3.1 XFMS program. The methods described here can generally be applied to determine the effects of external reagents on aggregation pathways, and can be used as both structural and kinetic assays to determine batch-to-batch variability. For Aβ aggregation in particular, these methods thus provide a robust approach to study pathways by combining high-resolution residue-specific insights with global structural changes across timepoints from seconds to hours.

## 8. Conclusions and future directions

The XFMS experiment has evolved substantially since its introduction in the late 1990s, starting from a setup as simple as exposing a single sample in a microfuge tube to an automated sample handling unit coupled with hybrid multimodal spectroscopy and microsecond radiolytic labeling, as described in this article. Other advances have included new ways of labeling proteins, such as with the recently reported multiplex labeling method using tri­fluoro­methyl­ation (Jain *et al.*, 2025 ▸). Each advance has provided new approaches for characterizing protein interactions and dynamics. The developments described here further advance the field in several ways. First, the beamline 3.3.1 developments make the XFMS field more accessible and adaptable to other synchrotron centers. Here we have shown some key strategies for endstation development in compact spaces, including adopting sample configurations with fast and efficient alignment for a microfocused beam and a micrometre-sized sample with high precision. Second, we demonstrated the utility of the versatile sample exposure conditions by automated Alexa488 dose-response analysis and discussed the pros and cons of the single exposure versus multiple sample exposure methods for analyzing protein–protein interactions. Third, we demonstrated the utility of the new hybrid spectroscopy-XFMS approach to monitor both global and local conformational changes during Aβ40 aggregation. This method offers a valuable tool for investigating the molecular mechanisms of higher-order structure formation and aggregation dynamics, with potential applications in therapeutic development. Fourth, we reported empirical dose measurements for the XFMS sample configuration, which is crucial for correlating absorbed dose with the exposure conditions, especially for radiation damage studies on biomolecules or cell samples. Future implementations include a fast shutter with translation stage to synchronize exposure times, enabling direct Alexa–Gafchromic dose correlation. The new inline setup enables real-time fluorescence monitoring of radiolysis under controlled dose rates and doses, and can be extended to track radical chemistry in organic and inorganic systems, which might be useful for screening radiosensitive drugs for radiotherapy. Future advances will also include microscopes for imaging-based monitoring of cellular samples during irradiation and downstream proteomics or cellular analysis. In summary, this dedicated West Coast XFMS facility is positioned to drive significant advances not only for the structural biology community but for a broad range of user groups. Researchers interested in exploring project opportunities can apply for beam time through the Molecular Foundry user portal (https://foundry.lbl.gov).

## Supplementary Material

Methods S1 and S2; Figs. S1 to S6; Tables S1 to S5. DOI: 10.1107/S1600577526003711/tv5086sup1.pdf
