# Supplementary material for: Design and commissioning of a new synchrotron beamline dedicated to X-ray footprinting mass spectrometry
Source: J Synchrotron Radiat. 2026 May 7;33(Pt 4):1206–18. doi: 10.1107/S1600577526003711 (PMC13344602; doi:10.1107/S1600577526003711)
Supplement: Supplementary file 1 [file s-33-01206-sup1.pdf]

JOURNAL OF SYNCHROTRON RADIATION

Volume 33 (2026)

Supporting information for article:

Design and commissioning of a new synchrotron beamline dedicated to X-ray footprinting mass spectrometry

Sayan Gupta, Brandon Russell, Line G. Kristensen, Jared de Chant, Anthony Lu, Lieselotte Obst-Huebl, Behzad Rad, James Tyler, Simruthi Subramanian, Savannah Kidd, Sathi Paul, Yan Chen, Christopher J. Petzold, Darren Kahan, Shawn M. Costello, Kei Nakamura, Jamie L. Inman, Alastair MacDowell, Adrian Spucces and Corie Y. Ralston

Method S1: Protein purification and preparation for XFMS

Method S2: Monitoring Aβ peptide aggregation in the presence of ThT by simultaneous fluorescence and XFMS

Figure S1: Raytrace of toroid mirror focus at beamline 3.3.1

Figure S2: Effect on flux density by moving sample downstream of mirror focus

Figure S3: Effect on flux density by lowering the sample at the mirror focus

Figure S4: Endstation software control panel

Figure S5: Error estimation for sample exposure using 200 μm ID capillary flow set-up

Figure S6: XFMS dose-response based analysis of the barstar-barnase complex

Table S1: Dose rate analysis by Gafchromic film dosimetry

Table S2: Dose rates as measured by Gafchromic film dosimetry at various vertical positions within the focused X-ray beam

Table S3: Rate constants of hydroxyl radical modification and the ratio between free barnase and the barstar-barnase complex

Table S4: Percentage (%) of hydroxyl radical modification and the ratio between free barnase and the barstar-barnase complex

Table S5: Fitting parameters for the Aβ +/– ThT hybrid fluorescence–XFMS kinetics

Method S1: Protein purification and preparation for XFMS

Purification of SpyCatcher003, complex formation with SpyTag001, and sample exposure

Briefly, BL21(DE3) harboring the pLysS plasmid was transformed with the pDEST14-SpyCatcher003 S49C vector (Addgene). Colonies were picked and grown in LB supplemented with 40 µg/mL Chloramphenicol and 100 µg/mL Ampicillin overnight at 37°C with 250 rpm shaking. The overnight cultures were used to inoculate 0.5 L LB media supplemented with 40 µg/mL Chloramphenicol and 100 µg/mL Ampicillin and grown to ~0.6 OD. The bacterial cells were induced by adding a final concentration of 0.4 mM IPTG to the media and growing the cultures at 30°C. The bacterial cells were harvested after 3 hours by centrifuging the cultures at 6000 rpm in a JLA8.10 fixed angle rotor centrifuge (Beckman Coulter) for 20 minutes at 4°C. Pellets were resuspended in lysis buffer (50 mM Tris pH 8.0, 300 mM NaCl, 10 mM Imidazole, 5% (v/v) glycerol) and lysed by two passages through an Emulsiflex C3 homogenizer at >20,000 PSI(Avestin). The lysate was spun down at 100000 x g in a Ti70 rotor. The clarified lysate was loaded onto a HiTrap FF Ni-NTA column, washed with lysis buffer, followed by lysis buffer containing 25 mM

imidazole for 5 column volumes, and then eluted with 250 mM imidazole step gradient. After extensive dialysis to remove imidazole, protein was flash frozen with liquid nitrogen and stored at -80°C. For XFMS, the protein samples are buffer exchanged in 10 mM sodium phosphate by size exclusion chromatography and diluted to ~ 10 μM concentration. Equimolar amounts of SpyCatcher003 and SpyTag001 (purchased from Anaspec Inc.) in 10 mM sodium phosphate buffer were mixed at room temperature for 30 min to form the SpyCatcher003-Spytag001 complex. Samples were exposed using both 75 μm jet and 200 μm ID capillary flow as indicated in the main text. Exposed samples were collected in a tube containing ~ 10 mM methionine amide to scavenge secondary radicals. Samples were desalted, digested with trypsin and AspN and analysed by LCMS following standard procedures.

Purification of barstar-barnase, and sample exposure

Plasmid encoding for either 6xHis-MBP-precission-barnase H102A D44C or 6xHis-MBP-precission-barstar C40A were transformed into Rosetta 2(DE3)pLysS cells for expression. For each plasmid, one liter of cells was induced at OD = 0.6 with 1mM IPTG and grown at 37 °C for 3 hours. Cells were then pelleted, resuspended in 50 mM HEPES pH 7.0, 150 mM NaCl, 0.5 mM TCEP, and lysed via sonication. Cell debris was then pelleted (ss34, 14k rpm, 30 minutes at 4 °C) and the resulting supernatant was filtered (0.22 μm). Clarified lysate was allowed to batch bind to HisPur Ni2+-NTA resin (Thermo Fisher Scientific) washed with 50 mM HEPES pH 7.0, 150 mM NaCl, 25 mM imidazole, 0.5 mM TCEP, and protein was then eluted with 50 mM HEPES pH 7.0, 150 mM NaCl, 500 mM imidazole and 0.5 mM TCEP. Prescission protease was added to the eluate and then dialyzed overnight into 50 mM HEPES pH 7.0, 150 mM NaCl, 0.5 mM TCEP. Cleaved protein was allowed to batch bind to HisPur Ni2+-NTA resin washed with 50 mM HEPES pH 7.0, 150 mM NaCl, 25 mM imidazole, 0.5 mM TCEP. The flowthrough containing either barnase H102A D44C or barstar C40A was collected, concentrated using a 3 kD cutoff spin concentrator (Amicon®) and further purified and buffer exchanged using a HiLoad 16/600 Superdex 75 pg column equilibrated with 25mM HEPES KOH pH 7.5, 15mM MgOAc, 150mM KCl, 0.1mM TCEP. Fractions containing either barnase H102A D44C or barstar C40A were pooled and glycerol was added to a final concentration of 5%. Samples were then flash frozen in liquid nitrogen and stored at -80C for future use. For XFMS, the protein samples are dialyzed against 10 mM sodium phosphate containing 100 mM NaCl and diluted to ~ 10 μM concentration. An equimolar concentration of barnase and barstar was mixed to form the complex. Samples were exposed using capillary flow as indicated in the main text. Exposed samples were collected in a tube containing ~ 10 mM methionine amide to scavenge secondary radicals. Samples were desalted, digested with trypsin, and analysed by LCMS following standard procedures. The dose response curves (fraction unmodified vs. X-ray exposure) were fitted to single exponential functions

in Origin® (OriginLabs). The rate constant, *k* (sec^{-1}), was used to measure the reactivity and solvent accessibility of side chains towards hydroxyl radical-induced modification. The reported errors of the rate data were determined by the Origin program using 95% confidence limits of the fitting results. The R^2 of the fits were between 0.98-0.99. The ratio of rate constants provided the relative change in the solvent accessibility between the free protein and the complex.

Preparation of Amyloid beta and Thioflavin T

Synthetic, hexafluoroisopropanol (HFIP)-treated Aβ40 (AS-64128) was purchased from AnaSpec (Fremont, CA). Peptide films were dissolved in 300 µL of 10 mM NaOH per 0.5 mg of Aβ40 peptide by incubating at ambient temperature for 5 min and brief vortexing, followed by bath sonication for 1 min at ambient temperature. Peptide stocks were stored on ice and used within 24 h. A 1:10 dilution of the peptide stock in ice-cold PBS (Gibco, pH 7.4) was used to determine the peptide concentration by UV absorbance at 214 nm on a NanoDrop 2000 spectrophotometer using an Aβ40 extinction coefficient of 91,462 (M^{-1} cm^{-1}) (Gunn et al. *J. Biol. Chem.* 2016, *291* (12), 6134–6145).

Thioflavin T (ThT, Sigma-Aldrich) was prepared as a 1 mM stock solution in MilliQ water and filtered through a 0.22 µm filter. ThT stock concentration was determined by absorbance at 412 nm using a PerkinElmer Lambda 365 UV-Vis spectrophotometer and an extinction coefficient of 36,000 (M^{-1} cm^{-1}).

Method S2 : Monitoring Aβ peptide aggregation in the presence of ThT by simultaneous fluorescence and XFMS

15 mL of 40 µM Aβ40 in PBS with and without 40 µM ThT were each prepared in a 50 mL conical tube immediately before the start of the Aβ40 aggregation assay. The two samples were incubated in an Eppendorf ThermoMixer set to 37°C, 400 rpm and aliquots for simultaneous X-ray footprinting fluorescence spectroscopy at beamline 3.3.1 were taken at indicated time-points. A single exposure of 750 µs was used for kinetics studies

The sample fluorescence probe or detection area was configured vertically immediately above the point of X-ray exposure. The approximate detection area is approximately 0.5 mm², with a 20X objective magnification (~10 mm light-gathering area divided by the objective magnification), which yields a fluorescence detection volume ~ 15.7 nl for the 200 µm ID capillary flow exposure setup. The LED light source and optical filters were set to provide an excitation wavelength of around 450 nm and collect emission at approximately 500 nm. We used PMT to detect changes in the emission intensity. LabVIEW-based software controlled the flow speed, exposure time, sample collection volumes, and spectroscopy data collection and storage.

Irradiated samples were collected in tubes containing L-Met-NH_2•HCl (10 mM final concentration) to prevent secondary reactions after exposure. Samples were stored at -80°C until overnight protease digestion at 37°C using trypsin/Lys-C (Promega, V5073) at a 1:20 (w/w) protease-to-protein ratio in 1 M urea, 50 mM ammonium bicarbonate. Digested samples were desalted using C18 spin columns (Pierce, 89870) prior to LCMS analysis which was performed as previously described (He et al. *J Mol Biol*. 2024 Aug 15;436(16):168650).

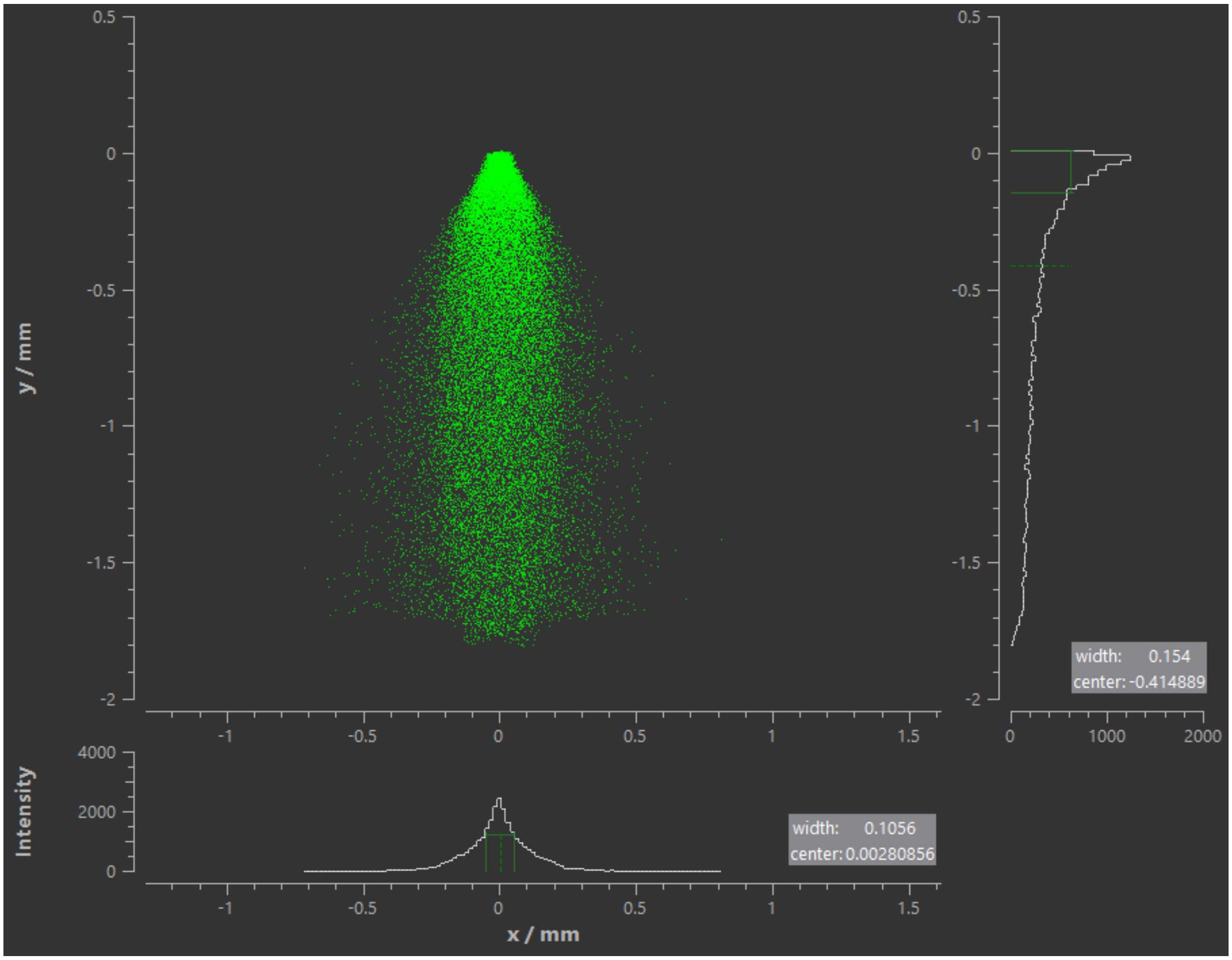

Figure S1. Raytrace of toroid mirror focus at beamline 3.3.1. The mirror is located at 12.3 m from the source, and calculations assumed ALS ring energy 1.9 GeV, an ALS bend magnet source, and X-ray energy 5 KeV. The source size is 94 ×16 μm FWHM (H x V) and the mirror accepts 3 x 0.5 mrad (H × V). The mirror toroid is not in the usual 1:1 optical configuration that minimizes optical aberrations. The mirror magnifies the source by × 0.4 with consequential increase in divergence at the focus of 7.5 x 1.2 mrad (H × V). The focus may be expected to be smaller than the source size due to the demagnification but the raytraced focus shown here in green is actually larger due to aberrations and indicates a focus spot size of 105 × 197 μm FWHM (H × V), with a significant tail in the vertical.

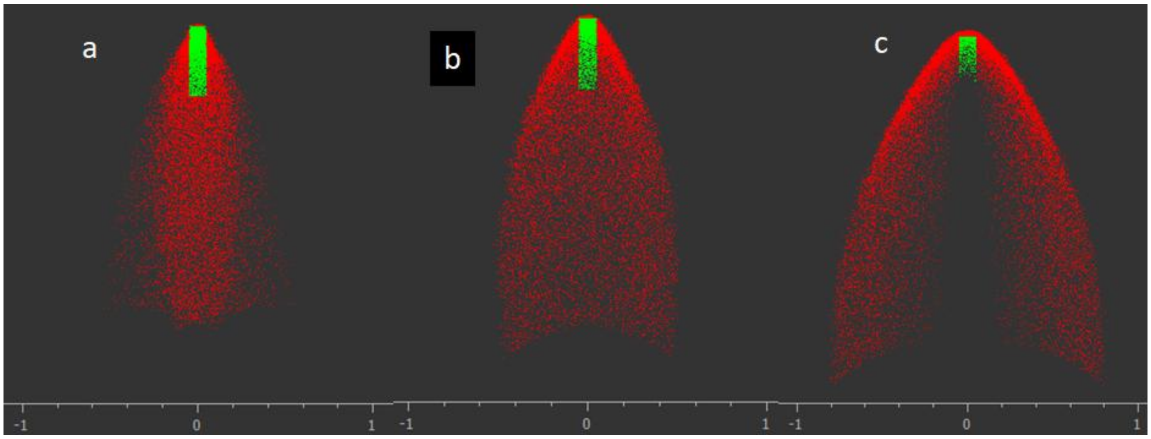

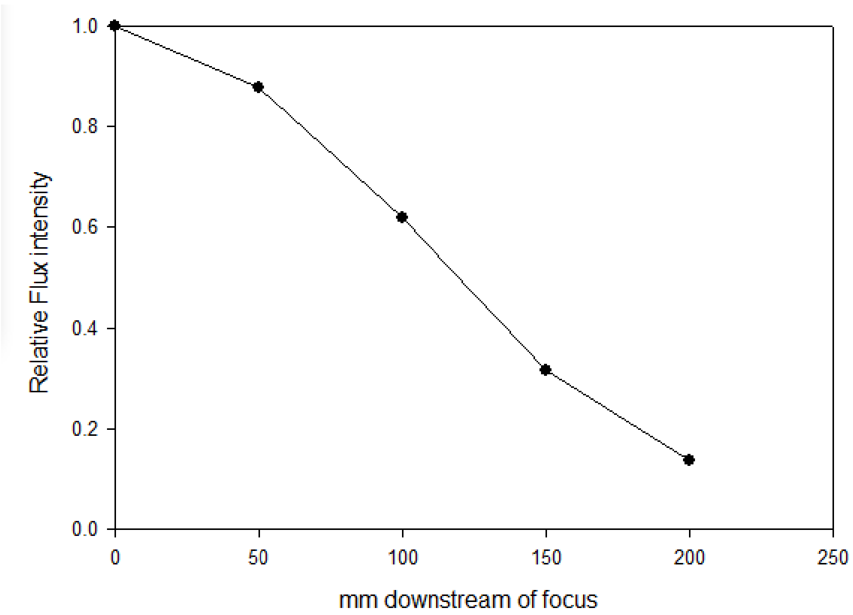

Figure S2. Effect on flux density by moving sample downstream of mirror focus. (Top) Ray trace of mirror focus (red) with sample area (100 µm width × 400 µm height, green), a) sample with max flux density (100%), b) sample 100 mm downstream of focus(62%) , c) sample 200 mm downstream of focus (13%). Note that for c) the sample area is not fully illuminated due to the particular beam structure at his defocused location. Scale in mm. **(Bottom)** Plot of calculated relative flux density in 100 × 400 µm^2 sample size versus distance downstream from focus.

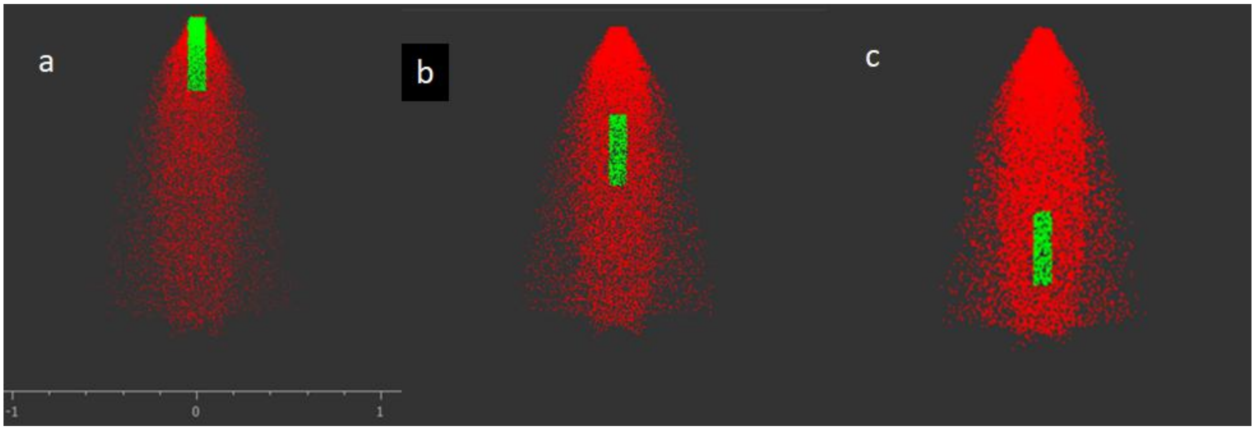

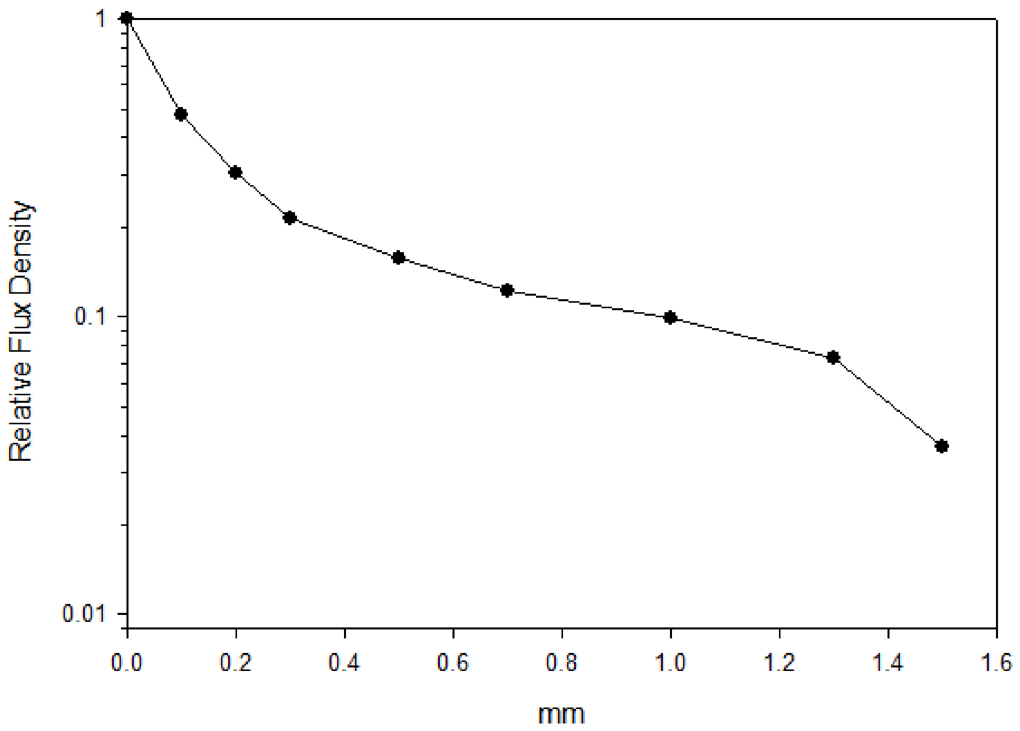

Figure S3. Effect on flux density by lowering the sample at the mirror focus. (Top) Ray trace of mirror focus (red) with sample area (100 µm width x 400 µm height, green), (a) sample with max flux density (100%), (b) sample 500 um lower (15%), (c) Sample 1 mm lower (10%). Scale in mm. When lowering the sample beyond 1.5mm the sample volume no longer starts to be illuminated by the coma tail. **(Bottom)** Plot of calculated relative flux density in 100 × 400 μm^2 sample size versus height below focus.

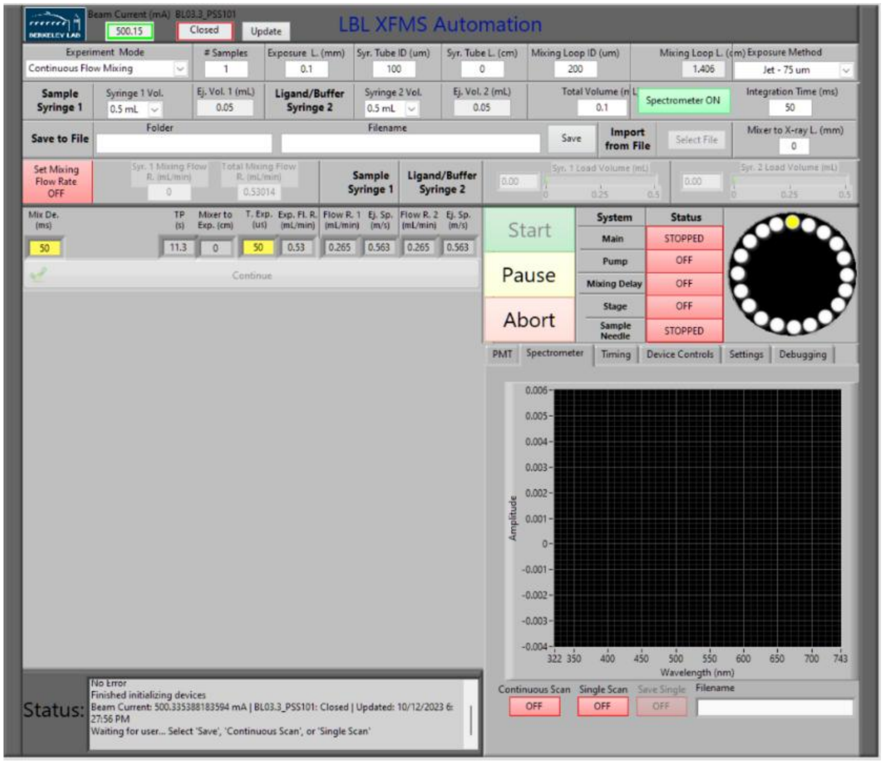

Figure S4. Endstation software control panel. Screenshot of the GUI experiment control panel. The configuration includes user input for the number of samples, target volumes, sample flow parameters, and syringe parameters. The control panel has three modes of operation – continuous mixing, stopped-flow mixing and steady-state flow for spectroscopy-XFMS data collection.

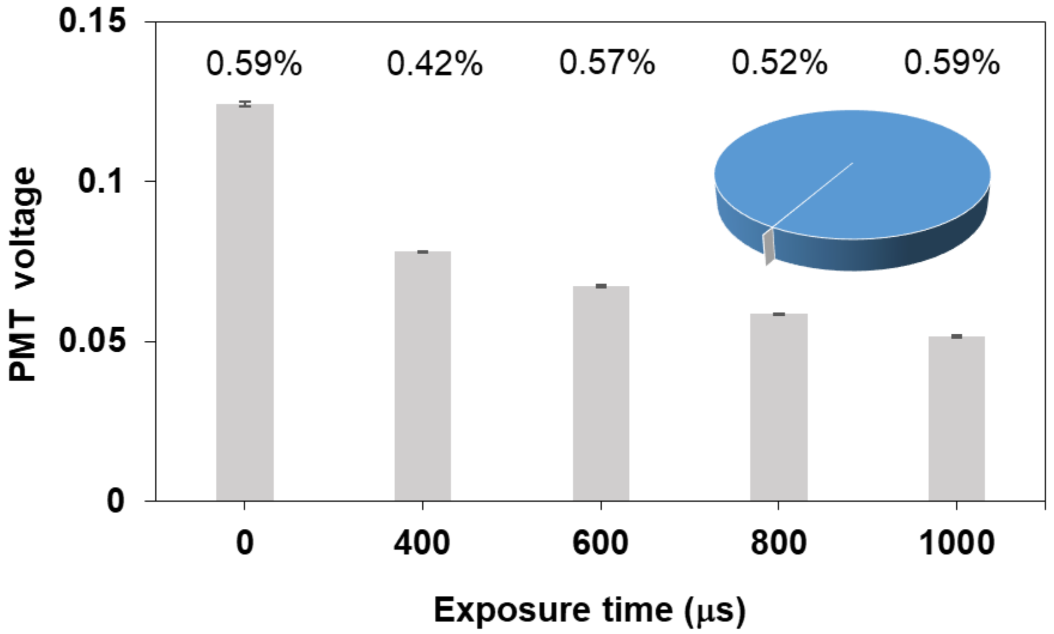

Figure S5. Error estimation for sample exposure using 200 μm ID capillary flow set-up. The bar plot shows the average photomultiplier tube (PMT) voltage generated from the emission of Alexa 488 after various exposure times as measured by automated inline fluorescence run in triplicates. The percentage values above the bar indicate variation of the standard error from the average PMT voltage. The inset provides an idea of the relative volume of the X-ray exposure window compared to the total volume of the sample for the XFMS experiment in general.

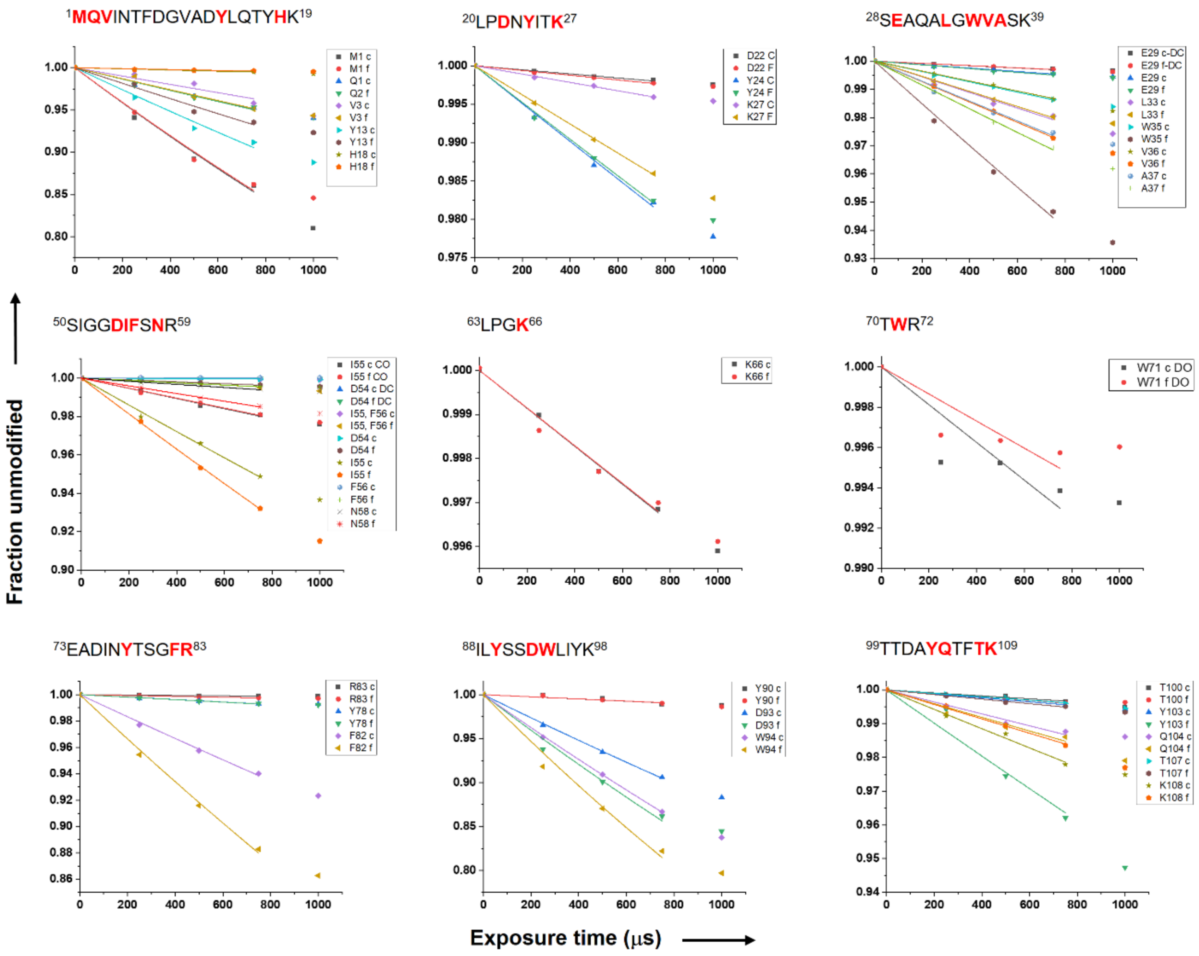

Figure S6. XFMS dose-response based analysis of the barstar-barnase complex. Residue specific dose response of barnase in the presence (c = complex) and absence (f = free) of barstar, collected using the capillary exposure system. Points are collected at a fixed dose rate for various exposure times, and fit to a single exponential fit (solid lines) to determine the hydroxyl radical reactivity k (s^{-1}), as described in the supporting methods S1 and results summarized in Table S3. Note that the last point is not included in the fit to exclude any non-linearity associated with secondary radical reactions / damage at the highest exposure.

Table S1. Dose rate analysis by Gafchromic film dosimetry

Exposure (s)[a]	1	2	3	4	5	6	7	8	9	10	11	12	13	14	15	16	17	18	19	20	21	22	23	24
**2 mm Aluminum[b]**																								
	**Dose rate (Gy / s) at each Pixel (82 X 82 μm²)[c]**																							
8.0	5.4	5.8	7.4	7.3	7.6	7.5	9.0	8.6	8.0	9.1	9.7	10.4	9.7	11.0	11.2	12.2	13.3	15.3	19.2	25.1	31.8	40.5	49.2	55.7
8.0	5.6	6.1	6.4	7.3	7.3	8.4	8.6	9.0	8.7	8.3	8.5	8.6	9.2	8.9	10.3	11.7	15.3	14.2	19.7	21.0	40.8	48.5	50.4	56.4
8.0	5.9	6.1	6.4	7.0	7.6	8.3	7.7	7.8	8.4	8.6	9.6	9.5	11.3	9.5	11.4	11.2	12.6	15.0	18.5	23.7	34.1	42.7	49.4	56.4
8.0	5.8	6.2	7.1	6.8	7.8	8.1	9.6	9.6	9.2	8.8	9.2	9.5	10.1	9.7	11.1	11.3	14.1	14.9	18.9	22.0	28.8	39.0	52.5	56.7
16.0	5.3	5.7	6.3	6.1	7.0	8.5	8.4	8.9	9.3	9.6	10.2	10.4	10.1	11.7	11.0	13.0	14.9	15.9	20.7	30.7	39.8	48.8	49.7	52.3
16.0	5.0	5.1	6.3	6.3	7.3	7.3	8.3	8.3	8.2	8.8	9.1	9.3	9.9	11.7	12.6	13.5	16.9	15.9	22.0	29.2	37.7	46.4	50.7	55.4
16.0	5.5	5.9	6.8	7.8	9.0	8.5	7.9	8.0	9.0	9.2	9.8	9.6	11.9	10.1	12.1	12.4	14.1	16.1	20.8	27.8	30.9	38.0	47.9	55.8
16.0	4.9	5.6	6.3	7.4	7.5	7.7	8.6	8.3	9.9	9.0	10.7	11.7	10.8	11.3	11.7	13.0	13.6	15.7	22.3	31.0	36.4	48.4	61.3	71.6
4.0	6.3	7.1	7.6	8.2	9.0	9.2	10.2	9.4	10.0	10.0	10.3	10.7	10.5	10.3	11.7	10.9	12.8	14.0	16.7	17.2	20.9	31.1	42.4	50.3
4.0	6.6	7.7	7.3	8.1	7.8	9.4	8.9	9.7	9.4	10.4	10.1	9.7	10.6	10.5	11.3	10.9	10.9	12.9	14.8	17.1	19.6	27.1	32.9	40.0
4.0	3.5	5.8	6.6	6.6	7.6	8.4	7.9	8.5	9.2	9.7	8.8	9.3	10.5	10.3	10.2	10.0	11.0	12.3	10.6	13.3	13.2	16.4	17.7	71.4
4.0	7.0	7.7	7.9	8.7	8.7	8.6	8.9	10.2	10.1	9.2	9.8	10.6	10.7	11.2	11.1	13.4	14.1	15.9	21.0	25.6	33.4	42.9	48.0	64.5
	Average dose rate (Gy / s)																							
	**5.6**	**6.2**	**6.9**	**7.3**	**7.9**	**8.3**	**8.7**	**8.9**	**9.1**	**9.2**	**9.6**	**9.9**	**10.4**	**10.5**	**11.3**	**12.0**	**13.6**	**14.8**	**18.8**	**23.6**	**30.6**	**39.1**	**46.0**	**57.2**
	Error[d]																							
	**0.9**	**0.8**	**0.6**	**0.8**	**0.7**	**0.6**	**0.7**	**0.7**	**0.7**	**0.6**	**0.7**	**0.8**	**0.7**	**0.9**	**0.7**	**1.1**	**1.7**	**1.2**	**3.3**	**5.7**	**8.6**	**9.9**	**11.1**	**8.7**
**1 mm Aluminum[b]**																								
0.5	83.1	85.0	96.5	141.9	105.6	103.5	98.4	107.7	115.9	111.3	128.1	122.6	115.1	131.1	149.6	155.4	171.4	222.0	285.3	391.2	507.4	589.8	730.0	847.5
0.5	73.9	89.4	99.2	95.0	100.4	101.1	108.5	109.1	107.8	108.9	113.3	121.1	137.5	143.7	134.4	162.2	184.6	204.9	237.7	349.8	435.7	546.9	711.7	768.4
0.5	83.7	86.7	105.0	97.8	114.3	101.2	108.3	109.8	107.3	114.1	115.4	127.8	132.5	138.4	142.4	152.0	166.9	200.9	245.9	400.9	554.9	642.7	678.1	851.4

0.05	0.05	0.1	0.1	0.1	0.1	**0.5 mm Aluminum**		Error[d]		Average dose rate (Gy / s)	0.25	0.25	0.25	0.25	1.0	1.0	1.0	1.0	0.5
855.0	863.1	876.8	892.6	792.6	720.8		**14.1**		**88.1**		106.7	106.2	108.5	104.7	85.0	75.5	77.1	78.1	74.8
854.3	872.7	882.6	913.4	913.8	750.0		**12.0**		**92.2**		101.7	105.0	114.7	108.6	87.1	84.5	81.0	78.1	84.0
949.1	887.7	1058.0	913.0	917.5	877.3		**11.7**		**101.3**		122.6	119.9	109.1	104.4	100.2	87.9	90.3	93.7	86.3
946.4	848.6	969.4	915.8	864.3	830.2		**15.1**		**109.0**		124.9	121.7	122.1	109.4	98.3	97.1	100.6	96.4	102.2
980.5	1042.8	965.7	897.7	927.8	935.6		**14.4**		**109.3**		127.4	121.4	134.3	112.9	102.0	89.5	91.8	94.3	117.3
1043.6	1035.1	971.6	947.1	947.0	945.5		**10.6**		**106.2**		116.8	121.9	116.4	113.6	95.1	94.7	96.4	94.6	119.2
1008.8	966.2	948.2	1013.0	965.6	1030.3		**10.4**		**112.4**		128.5	120.6	132.5	118.4	104.3	107.5	106.4	111.5	104.4
950.4	1085.9	1061.1	1019.2	1027.8	999.3		**12.6**		**111.3**		128.7	131.8	124.7	119.3	102.1	93.8	102.2	94.7	112.2
1102.6	1038.8	1041.8	1064.1	1163.2	935.6		**15.7**		**113.7**		117.9	158.1	121.6	113.8	103.7	98.1	102.0	103.5	114.8
1074.5	1170.0	1153.4	1285.8	1196.7	1026.4		**9.7**		**117.2**		130.8	128.4	133.3	119.7	123.2	110.4	104.1	109.4	112.3
1123.1	1220.4	1164.8	1226.8	1110.5	1101.0		**12.9**		**123.6**		149.3	140.5	133.2	124.0	126.6	105.0	123.1	112.4	112.6
1277.8	1256.1	1366.4	1171.8	1275.1	1088.5		**9.6**		**130.2**		147.5	143.2	142.6	127.8	132.3	131.2	122.8	117.9	125.9
1275.6	1139.9	1424.2	1438.3	1472.9	1284.8		**13.5**		**132.6**		146.0	160.0	138.7	137.5	125.2	121.1	140.3	114.5	122.4
1449.6	1313.8	1620.1	1652.1	1528.0	1302.5		**7.5**		**141.5**		146.6	154.2	142.6	147.6	147.4	128.4	136.5	145.1	136.4
1496.6	1380.2	1842.9	1739.7	1666.8	1594.9		**7.5**		**148.6**		150.9	158.8	158.2	145.8	152.6	146.9	155.7	149.5	138.4
1715.3	1660.8	2013.7	1968.1	1713.2	1679.7		**14.7**		**164.8**		175.8	174.1	156.8	169.3	166.7	157.3	202.0	160.6	145.9
2081.9	1711.8	2346.6	2041.9	2073.7	1751.1		**16.2**		**187.6**		164.7	179.0	204.2	184.3	216.9	196.2	206.5	189.9	187.2
2422.7	2123.4	2776.1	2645.3	2194.8	2068.1		**23.5**		**219.1**		189.9	214.3	249.2	221.0	266.0	221.5	233.8	222.1	182.9
3291.8	2891.9	4279.5	3860.5	2978.8	2425.4		**35.3**		**262.0**		227.0	252.5	256.2	227.5	330.1	257.8	308.3	296.2	219.6
3375.6	3762.9	5122.7	5297.9	3896.7	3000.2		**50.0**		**353.8**		293.5	339.5	334.5	280.1	445.1	336.5	363.9	407.1	302.9
3899.0	4438.7	6440.7	6399.1	5084.3	4064.5		**72.5**		**463.4**		312.4	459.8	467.2	403.5	468.3	411.9	592.1	494.1	453.7
3647.3	6565.8	6533.5	6261.1	6118.5	5475.1		**81.7**		**555.5**		391.7	573.1	493.1	469.2	630.3	511.1	682.9	599.1	535.4
4572.6	7657.6	7202.2	6998.4	7552.9	6655.1		**77.9**		**633.2**		443.4	643.7	547.8	588.5	639.4	646.0	691.1	633.1	645.5
5070.4	8676.7	8422.1	7750.8	8190.8	7434.5		**110.1**		**747.3**		514.7	792.2	553.0	772.8	765.1	729.4	872.4	726.9	773.9

0.05	0.05	0.025	0.025	0.025	0.025	Average dose rate (Gy / s)	Error[d]
753.7	875.6	907.4	958.1	1076.8	1022.2	**882.9**	102.6
948.5	1001.2	1091.2	1039.5	1137.1	1192.0	**966.4**	129.1
988.4	907.9	1089.9	1052.6	1073.4	1162.8	**989.8**	94.4
1027.6	983.2	1152.5	1197.1	1122.3	1216.9	**1006.2**	136.8
999.3	1037.3	1234.8	1164.3	1103.5	1140.8	**1035.8**	105.2
995.6	1099.0	1135.7	1174.5	1181.4	1300.6	**1064.7**	114.5
1020.5	1002.1	1180.1	1064.8	1369.1	1195.9	**1063.7**	124.0
1121.2	1039.0	1203.0	1109.0	1159.7	1193.4	**1080.8**	79.0
1097.5	1142.1	1248.9	1158.2	1192.0	1734.4	**1159.9**	198.8
1061.7	1176.3	1246.4	1247.4	1265.3	1324.2	**1185.7**	94.2
1231.2	1203.9	1381.6	1240.0	1323.4	1392.2	**1226.6**	97.9
1304.6	1267.3	1379.3	1340.2	1363.1	1340.0	**1285.9**	85.7
1352.1	1344.9	1412.3	1316.7	1399.0	1470.5	**1360.9**	96.7
1346.8	1496.4	1427.5	1400.7	1730.9	1602.4	**1489.2**	140.0
1473.2	1667.0	1605.7	1586.3	1619.5	1689.8	**1613.5**	124.0
1900.0	1739.2	1656.8	1682.9	1814.0	1979.3	**1793.6**	135.6
1950.4	1985.9	1931.6	1831.2	1802.6	1947.9	**1954.7**	173.7
2167.6	2649.8	2397.3	1946.4	2085.4	2291.9	**2314.1**	265.4
2729.4	3320.0	3521.9	2362.8	3094.7	2870.5	**3135.6**	561.2
3274.7	3636.0	3596.6	3084.2	3470.3	3662.6	**3765.0**	725.7
4223.0	4098.2	5460.2	3221.9	5474.0	4807.7	**4800.9**	1000.9
5279.0	5547.7	6085.2	4171.8	5129.2	5330.6	**5512.1**	900.6
6827.0	5677.3	6189.7	4618.4	6589.4	5920.5	**6371.8**	1022.3
7512.9	7928.7	7177.9	5121.4	7267.6	9192.6	**7478.9**	1262.8

[a] X-ray exposures were set by using the Uniblitz® electronic shutter mounted downstream of the Be exit window.
[b] Aluminum sheets were mounted between the Be exit window and the sample.
[c] Corrected dose rates were calculated as described in the main text.
[d] Standard error.

Table S2. Dose rates as measured by Gafchromic film dosimetry at various vertical positions within the focused X-ray beam.

	0.5 mm Al[a]		1 mm Al		2 mm Al		0.5 mm Al	1 mm Al	2 mm Al	Average[f]	Error
Distance[b]	Av. Dose rate[c]	Error[d]	Av. Dose rate	Error	Av. Dose rate	Error	Normalized Av. dose rate[e]	Normalized Av. dose rate[e]	Normalized Av. dose rate[e]		
40.0	7478.9	1262.8	747.3	110.1	57.2	8.7	8.5	8.5	10.3	9.1	1.0
120.0	6371.8	1022.3	633.2	77.9	46.0	11.1	7.2	7.2	8.3	7.6	0.6
200.0	5512.1	900.6	555.5	81.7	39.1	9.9	6.2	6.3	7.0	6.5	0.4
280.0	4800.9	1000.9	463.4	72.5	30.6	8.6	5.4	5.3	5.5	5.4	0.1
360.0	3765.0	725.7	353.8	50.0	23.6	5.7	4.3	4.0	4.2	4.2	0.1
440.0	3135.6	561.2	262.0	35.3	18.8	3.3	3.6	3.0	3.4	3.3	0.3
520.0	2314.1	265.4	219.1	23.5	14.8	1.2	2.6	2.5	2.7	2.6	0.1
600.0	1954.7	173.7	187.6	16.2	13.6	1.7	2.2	2.1	2.5	2.3	0.2
680.0	1793.6	135.6	164.8	14.7	12.0	1.1	2.0	1.9	2.1	2.0	0.1
760.0	1613.5	124.0	148.6	7.5	11.3	0.7	1.8	1.7	2.0	1.8	0.2
840.0	1489.2	140.0	141.5	7.5	10.5	0.9	1.7	1.6	1.9	1.7	0.1
920.0	1360.9	96.7	132.6	13.5	10.4	0.7	1.5	1.5	1.9	1.6	0.2
1000.0	1285.9	85.7	130.2	9.6	9.9	0.8	1.5	1.5	1.8	1.6	0.2
1080.0	1226.6	97.9	123.6	12.9	9.6	0.7	1.4	1.4	1.7	1.5	0.2
1160.0	1185.7	94.2	117.2	9.7	9.2	0.6	1.3	1.3	1.7	1.4	0.2
1240.0	1159.9	198.8	113.7	15.7	9.1	0.7	1.3	1.3	1.6	1.4	0.2
1320.0	1080.8	79.0	111.3	12.6	8.9	0.7	1.2	1.3	1.6	1.4	0.2
1400.0	1063.7	124.0	112.4	10.4	8.7	0.7	1.2	1.3	1.6	1.3	0.2
1480.0	1064.7	114.5	106.2	10.6	8.3	0.6	1.2	1.2	1.5	1.3	0.2
1560.0	1035.8	105.2	109.3	14.4	7.9	0.7	1.2	1.2	1.4	1.3	0.1
1640.0	1006.2	136.8	109.0	15.1	7.3	0.8	1.1	1.2	1.3	1.2	0.1
1720.0	989.8	94.4	101.3	11.7	6.9	0.6	1.1	1.1	1.2	1.2	0.1
1800.0	966.4	129.1	92.2	12.0	6.2	0.8	1.1	1.0	1.1	1.1	0.0
1880.0	882.9	102.6	88.1	14.1	5.6	0.9	1.0	1.0	1.0	1.0	0.0

[a] Aluminum sheets were mounted between the Be exit window and the sample.
[b] Distance (μm) from the starting point of the focused beam spot to the center of each 80 μM pixel. The total distance accounted for 24 pixels, which was used for the calculation of dose.
[c] Average dose rate from Table S1.
[d] Standard error.
[e] Normalizations were done by dividing all dose rates by the lowest dose rate determined at position 1880 μm.
[f] Average of the normalized dose rate for 0.5, 1 mm, and 2 mm aluminum attenuation.

Table S3. Rate constants of hydroxyl radical modification and the ratio between free barnase and the barstar-barnase complex

Sequence of the peptide fragments[a]	Sites of modification[b]	Type of modification '(Da)[c]	Hydroxyl radical reactivity rate				R^e	R_{max}^e	R_{min}^e	R_{av}^e	Standard error
			Complex		Barnase						
			k (s^{-1})[d]	Error	k (s^{-1})[d]	Error					
GPG[1]MQVINTF DGVADYLQTY HK[19]	M1	+16	212.15	9.28	209.69	8.62	1.01	1.10	0.93	1.01	0.07
	Q2	+16	67.08	2.65	68.08	3.36	0.99	1.08	0.90	0.99	0.07
	V3	+16	50.02	5.68	66.13	3.93	0.76	0.90	0.63	0.76	0.11
	Y13	+16	132.55	6.88	93.26	5.53	1.42	1.59	1.27	1.43	0.13
	H18	+16	6.97	0.03	5.49	0.51	1.27	1.40	1.16	1.28	0.10
[20]LPDNYITK[27]	D22	+16	2.65	0.12	3.08	0.09	0.86	0.93	0.80	0.86	0.05
	Y24	+16	24.74	0.68	24.02	0.54	1.03	1.08	0.98	1.03	0.04
	K27	+16	5.43	0.12	19.03	0.15	0.29	0.29	0.28	0.29	0.01
[28]SEAQALGWV ASK[39]	E29	-30	4.04	0.23	3.96	0.18	1.02	1.13	0.92	1.02	0.09
	E29	+16	6.14	0.27	6.64	0.30	0.92	1.01	0.85	0.93	0.07
	L33	+16	27.95	1.37	27.03	0.32	1.03	1.10	0.97	1.03	0.05
	W35	+16	18.45	0.28	76.14	2.39	0.24	0.25	0.23	0.24	0.01
	V36	+16	17.77	0.29	36.54	0.28	0.49	0.50	0.47	0.49	0.01
	A37	+16	35.69	1.50	42.65	0.79	0.84	0.89	0.79	0.84	0.04
[50]SIGGDIFSNR[59]	I55	+14	26.76	0.76	26.19	0.72	1.02	1.08	0.97	1.02	0.05
	D54	-30	0.03	0.00	0.61	0.03	0.06	0.07	0.05	0.06	0.01
	I55,F56	+16	0.53	0.26	6.03	0.57	0.09	0.14	0.04	0.09	0.04
	D54	+16	0.71	0.03	4.86	0.09	0.15	0.15	0.14	0.15	0.01
	I55	+16	70.62	1.80	94.19	0.71	0.75	0.77	0.73	0.75	0.02
	F56	+14	0.00	0.00	5.99	0.22	0.00	0.00	0.00	0.00	0.00
	N58	+16	8.13	0.22	20.20	0.32	0.40	0.42	0.39	0.40	0.01
[63]LPGK[66]	K66	+16	4.32	0.10	4.29	0.24	1.01	1.09	0.93	1.01	0.07
[70]TWR[72]	W71	+32	9.39	1.59	6.73	1.17	1.39	1.98	0.99	1.45	0.41
[73]EADINYTSGF R[83]	R83	+16	1.49	0.14	3.27	0.06	0.46	0.51	0.40	0.46	0.04
	Y78	+16	9.30	0.37	9.43	0.26	0.99	1.05	0.92	0.99	0.05
	F82	+16	84.46	1.82	170.70	3.65	0.49	0.52	0.47	0.50	0.02
[88]ILYSSDWLIYK[98]	Y90	+16	12.37	2.12	12.24	1.24	1.01	1.32	0.76	1.03	0.23
	D93	+16	133.32	1.41	206.69	9.09	0.65	0.68	0.61	0.65	0.03
	W94	+16	191.30	1.39	273.27	12.88	0.70	0.74	0.66	0.70	0.03
[99]TTDAYQTFTK[109]	T100	+16	4.41	0.33	5.20	0.14	0.85	0.94	0.77	0.85	0.07
	Y103	+16	5.88	0.12	49.49	4.36	0.12	0.13	0.11	0.12	0.01
	Q104	+16	17.83	0.98	20.42	1.58	0.87	1.00	0.77	0.88	0.10
	T107	+16	5.08	0.17	6.82	0.24	0.74	0.80	0.69	0.75	0.04
	K108	+16	28.89	1.00	21.55	0.47	1.34	1.42	1.27	1.34	0.06

[a] sequences of tryptic fragments used for the identification and quantification of modification sites.

[b] modified residues, which were identified and confirmed by LCMS/MS.

[c] type of side chain modification includes hydroxylation, carbonylation and decarboxylation, and dioxidation which resulted in a mass shift of +16, +14, -30 and + 32 Da.

[d] hydroxyl radical rate constants were estimated by employing a first-order exponential fit of the dose response plot as described in **S1** and **Figure S7**. The modified peptide fragments were eluted as a single peak or multiple peaks.

[e] ratio of hydroxyl radical reactivity obtained by dividing hydroxyl radical reactivity rate of barnase in the barstar-barnase complex by that of barnase when free. The ratio R represents a fold decrease (<1) or increase (>1) in the solvent accessibility of the modified residues. R_{max} and R_{min} indicated the highest and the lowest values estimated from the error of the reactivity rate. R_{av} represents the average of R, $R_{max,}$ and R_{min}.

Table S4. Percentage (%) of hydroxyl radical modification and the ratio between free barnase and the barstar-barnase complex.

Sequence of the peptide fragments[a]	Sites of modification[b]	Type of modification(Da)[c]	Ratio of % modification Complex / barnase[d]				R_{av}	Standard error
			250 μs	500 μs	750 μs	1000 μs		
[1]MQVINTFDGVADYLQTYHK[19]	M1	+16	1.11	0.99	1.01	1.23	1.09	0.10
	Q2	+16	0.98	0.95	1.01	1.02	0.99	0.03
	V3	+16	0.78	0.57	0.86	1.01	0.80	0.16
	Y13	+16	1.84	1.37	1.36	1.45	1.51	0.20
	H18	+16	0.90	1.11	1.32	1.44	1.19	0.21
[20]LPDNYITK[27]	D22	+16	0.89	0.98	0.86	0.94	0.92	0.05
	Y24	+16	0.99	1.07	1.01	1.10	1.04	0.05
	K27	+16	0.42	0.33	0.33	0.30	0.35	0.04
[28]SEAQALGWVASK[39]	E29	-30	0.94	1.20	0.97	0.93	1.01	0.11
	E29	+16	0.99	0.88	0.94	0.95	0.94	0.04
	L33	+16	1.16	1.10	0.98	1.17	1.10	0.07
	W35	+16	0.24	0.23	0.26	0.25	0.25	0.01
	V36	+16	0.63	0.56	0.55	0.58	0.58	0.03
	A37	+16	0.99	0.87	0.84	0.79	0.88	0.07
[54]SIGGDIFSNR[59]	I55	+14	0.86	1.08	1.00	1.03	0.99	0.08
	D54	-30	0.05	0.05	0.08	0.09	0.07	0.01
	I55,F56	+16	0.81	0.31	0.29	0.26	0.42	0.23
	D54	+16	0.12	0.13	0.16	0.17	0.15	0.02
	I55	+16	0.89	0.73	0.75	0.75	0.78	0.07
	F56	+14	0.03	0.04	0.03	0.02	0.03	0.01
	N58	+16	0.46	0.42	0.41	0.38	0.42	0.03
[63]LPGK[66]	K66	+16	0.81	1.00	1.04	1.05	0.98	0.10
[70]TWR[72]	W71	+32	1.17	1.13	1.21	1.32	1.21	0.07
[73]EADINYTSGFR[83]	Y78	+16	0.84	0.68	0.56	0.61	0.68	0.11
	F82	+16	1.10	0.95	0.98	0.90	0.98	0.07
	R83	+16	0.51	0.51	0.51	0.56	0.52	0.02
[88]ILYSSDWLIYK[98]	Y90	+16	0.54	0.74	1.11	0.88	0.82	0.21
	D93	+16	0.61	0.69	0.70	0.77	0.69	0.06
	W94	+16	0.63	0.72	0.76	0.81	0.73	0.07
[99]TTDAYQTFTK[109]	T100	+16	1.05	0.68	0.91	1.36	1.00	0.25
	Y103	+16	0.30	0.12	0.12	0.11	0.16	0.08
	Q104	+16	0.69	0.91	0.89	0.66	0.79	0.11
	T107	+16	0.71	0.62	0.81	0.83	0.74	0.08
	K108	+16	1.50	1.21	1.32	1.08	1.28	0.15

Table Footnotes

[a] sequences of tryptic fragments used for the identification of quantification of modification sites.

[b] modified residues, which were identified and confirmed by LCMS/MS.

[c] type of side chain modification includes hydroxylation, carbonylation and decarboxylation, and dioxidation which resulted in a mass shift of +16, +14, -30 and + 32 Da.

[d] The modified peptide fragments were eluted as a single peak or multiple peaks. The % modification is calculated relative to the native/unmodified peptide at different exposure times. The ratio is determined from % modification for barnase in the barstar-barnase complex divided by that of barnase when free.

[e] R_{av} represents the average of R for all the exposure points.

Table S5: Fitting parameters for the Aβ +/– ThT hybrid fluorescence–XFMS kinetics

Residue	ThT	t_{50}[3] (min)	*k*[3] (min^{-1})	m_1[3] (min^{-1})	m_2[3] (min^{-1})	A_1[3]	A_2[3]	R^2 [3]
500 nm[1]	+[4]	91.07184 ± 1.94289	-8.26285 ± 1.44681	0.00269 ± 1.48065E-4	-0.0043 ± 7.3657E-4	1.0374 ± 0.03386	1.04746 ± 0.02848	0.99507
E3[2]	–[5]	90 ± 0	87.74598 ± 42.24821	7.02976E-4 ± 4.40749E-4	7.60166E-5 ± 1.11183E-4	0.17337 ± 0.03804	0.01 ± 0	0.69924
E3	+	89.31467 ± 14.58915	24.21398 ± 9.03858	0.00118 ± 5.1744E-4	-1.8017E-4 ± 1.09008E-4	0.16801 ± 0.00991	0.15994 ± 0.02976	0.95491
F4	–	100 ± 0	10.97924 ± 4.6443	-1.73811E-5 ± 0.00169	-0.00124 ± 6.01596E-4	1.53514 ± 0.08058	0.82888 ± 0.13894	0.95482
F4	+	76.92325 ± 6.10653	15.91982 ± 3.36416	0.00629 ± 0.00231	-0.00111 ± 2.83932E-4	1.06981 ± 0.0441	0.72454 ± 0.06694	0.9841
H6,V12	–	100 ± 15.52872	15.65831 ± 10.74737	0.00274 ± 0.0027	-0.0012 ± 6.79402E-4	0.85729 ± 0.08339	0.72592 ± 0.17133	0.89676
H6,V12	+	95.57186 ± 4.33062	6.33247 ± 3.46558	0.00175 ± 0.00145	-0.00105 ± 3.09964E-4	0.89676 ± 0.05914	0.68242 ± 0.06998	0.95012
Y10, E11	–	100 ± 23.36773	20 ± 14.9594	0.00247 ± 0.00344	-5.33891E-4 ± 8.08082E-4	0.63135 ± 0.08201	0.39278 ± 0.21274	0.85837
Y10, E11	+	110 ± 0	10 ± 0	9.60081E-4 ± 4.70512E-4	-8.90515E-4 ± 1.85057E-4	0.3519 ± 0.02955	0.4624 ± 0.04213	0.84584
H13, H14	–	93.69248 ± 11.10418	21.73506 ± 6.79438	0.03044 ± 0.0152	-0.00613 ± 0.00315	4.56983 ± 0.31036	2.99195 ± 0.83892	0.97227
H13, H14	+	91.28275 ± 3.8208	9.88339 ± 2.73681	0.01966 ± 0.00752	-0.00706 ± 0.00143	4.74551 ± 0.26567	3.53932 ± 0.33209	0.97879
L17	–	77.61896 ± 6.67789	19.09539 ± 3.49113	0.00568 ± 0.00174	-7.90513E-4 ± 2.12615E-4	0.66136 ± 0.02813	0.42861 ± 0.05365	0.98869
L17	+	102.25024 ± 5.93799	10.4489 ± 4.31481	-4.78931E-4 ± 5.4868E-4	-7.98151E-4 ± 1.32168E-4	0.61175 ± 0.02151	0.44162 ± 0.0316	0.9825
V18	–	98.37714 ± 5.69716	13.00426 ± 3.9437	6.55991E-4 ± 1.99236E-4	-2.24825E-4 ± 4.50211E-5	0.10896 ± 0.00614	0.10377 ± 0.01105	0.98
V18	+	100 ± 0	84.64 ± 26.85713	2.68425E-4 ± 2.51037E-4	-7.80515E-5 ± 1.09124E-4	0.09 ± 0	0.04709 ± 0.03224	0.84656
F19.F20	–	84.29455 ± 5.1576	16.00904 ± 2.84994	0.0205 ± 0.00626	-0.00468 ± 8.98395E-4	2.96906 ± 0.12959	1.96019 ± 0.22134	0.99021
F19,F20	+	98.39422 ± 3.45165	11.11587 ± 2.44922	1.2662E-4 ± 0.00196	-0.00372 ± 4.23376E-4	2.62037 ± 0.07082	1.76044 ± 0.10122	0.99296
E22	–	73.57864 ± 26.58209	27.86712 ± 13.3164	0.00262 ± 0.0021	-3.31683E-4 ± 2.58093E-4	0.25153 ± 0.02377	0.17754 ± 0.07197	0.95611
E22	+	112.34322 ± 12.74796	12.0971 ± 9.78091	-7.98065E-5 ± 2.14811E-4	-3.29562E-4 ± 6.98464E-5	0.1939 ± 0.00927	0.17444 ± 0.01744	0.96251
D23	–	95.99401 ± 4.82413	10.14356 ± 3.46121	2.5133E-4 ± 1.03823E-4	-1.1556E-4 ± 2.58489E-5	0.07488 ± 0.00384	0.06371 ± 0.00616	0.97336

D23	+	104.31865 ± 8.83712	11.12902 ± 6.50877	9.86718E-5 ± 6.55409E-5	-1.01313E-4 ± 1.87307E-5	0.05238 ± 0.00277	0.05497 ± 0.00457	0.95937
V24	–	127.98758 ± 29.8467	27.07438 ± 14.91781	9.7815E-4 ± 0.00125	-1.6328E-4 ± 0	0.27859 ± 0.03717	0.13832 ± 0.02533	0.83729
V24	–	110.09213 ± 57.06992	43.05868 ± 19.45003	0.001 ± 0.00107	-8.05016E-5 ± 5.30469E-5	0.16416 ± 0.01696	0.1 ± 0	0.85565
N27, K28	+	93.60774 ± 5.01227	10.21695 ± 3.60318	0.00223 ± 0.00101	-9.49321E-4 ± 2.22794E-4	0.53568 ± 0.03596	0.41767 ± 0.05208	0.96808
N27, K28	–	96.93892 ± 4.12804	8.4266 ± 3.16255	0.00122 ± 4.45269E-4	-7.06794E-4 ± 1.05616E-4	0.33881 ± 0.01861	0.33027 ± 0.02458	0.974

Table Footnotes

[1] fluorescence emission with a 10 nm bandpass filter at 500 nm

[2] amino-acid residue modifications identified and quantified by LC-MS/MS analysis. Modified residues E3 and F4 were identified from peptide [1]DAEFR[5], modified residues H6, Y10, H13 were identified from peptide [6]HDSGYEVHHQK[16], modified residues L17, V18, F19, F20, E22, D23, V24, N27 and K28 were identified from peptide [17]LVFFAEDVGSNK[28], and modified residue M35 was identified from peptide [29]GAIIGLMVGGVV[40]. Data point at 135 min showed a large shift towards high value of % modification for most of the residues (except N27, K28) identified from the peptide [7]LVFFAEDVGSNK[28] , and hence, it was not included during the fitting iterations.

[3] transition midpoint (t_{50}), rate of the sigmoidal transition (k), slope (m_1) of change of initial level of % modification (A_1) before transition, slope (m_2) of change of final level of % modification (A_1) before transition and goodness of the fit (R^2)

[4,5] presence and absence of ThT

M35 data was not fitted.

Fitting equation: All data points obtained by tracking the fluorescence intensity of thioflavin-T during Ab40 protein aggregation and site-specific modification identified and quantified by LCMS/MS are fitted with the following modified Boltzmann sigmoidal equation:

$$y = (A_2 + m_2x) + ((A_1 + m_1x) - (A_2 + m_2x) / (1 + exp((x-t_{50}) /k))$$
